# A national study of Veterans with major upper limb amputation: Survey methods, participants, and summary findings

**DOI:** 10.1371/journal.pone.0213578

**Published:** 2019-03-14

**Authors:** Linda Resnik, Sarah Ekerholm, Matthew Borgia, Melissa A. Clark

**Affiliations:** 1 Research Department, Providence VA Medical Center, Providence, Rhode Island, United States of America; 2 Health Services, Policy and Practice, Brown University, Providence, Rhode Island, United States of America; 3 University of Massachusetts Medical school, Worcester Massachusetts, United States of America; Holland Bloorview Kids Rehabilitation Hospital, CANADA

## Abstract

**Introduction:**

A comprehensive study to assess quality and outcomes of care for Veterans with upper limb amputation is needed. This paper presents methods and summary findings from a national survey of Veterans with upper limb amputation.

**Methods:**

After completion of a pilot study to develop and refine methods, computer-assisted telephone interviews were conducted with 808 Veterans with upper limb amputation (response rate = 47.7%; cooperation rate = 63.3%).

**Results:**

Respondents were 776 unilateral and 32 bilateral amputees, 97.5% male, mean age 63.3 (sd 14.1). Prostheses were used by 60% unilateral and 91% bilateral, the majority used body powered devices. Prostheses were used ≥8 hours/day by 52% unilateral and 76% bilateral. Prosthetic training was received by 71% unilateral and 59% bilateral. Mean prosthetic satisfaction was 3.9 (sd 0.6) and 3.8 (sd 0.7) as measured by TAPES; and 25.0 (sd 5.1) and 25.7 (sd 4.5) as measured by OPUS CSD for unilateral and bilateral respectively. Mean perceived disability (measured by QuickDASH) scores were 49.5 (sd 20.7) for unilateral and 34.7 (sd 22.0) for bilateral. VR-12 PCS scores were below population norms. The majority reported contralateral limb pain, musculoskeletal conditions, back and neck pain. Phantom limb pain was reported in 83.4% of unilateral and 68.8% of bilateral, and residual limb pain in 65.1% of unilateral and 68.8% of bilateral. Most, (81.8% unilateral, 84.4% bilateral) had been to a Veterans Affairs medical center (VA) for amputation care, while 57% of unilateral and 81.3% of bilateral had been to a VA amputation clinic.

**Discussion/Conclusion:**

Veterans with upper limb amputation have moderately impaired physical functioning. Prosthesis use rates were lower than previously reported. Although satisfied with their prostheses, nearly half used them ≤8 hours/day. Rates of musculoskeletal problems, phantom and residual limb pain were higher than previously reported. A substantial proportion never received prosthetic training, or VA amputation care.

## 1.0—Introduction

Appropriate provision of upper limb prostheses and rehabilitation services can improve satisfaction with the prosthetic limb, reduce device abandonment and improve overall quality of life.[1] Regular use of a prosthesis may also prevent cumulative trauma disorders (CTD) in the sound side limb, as well as back and neck pain related to poor compensatory strategies, common problems of upper limb amputees.[2, 3] Limited research shows that prosthesis use is associated with improved performance in hygiene, grooming and dressing.[4] In contrast, non-use of a prosthesis is associated with development of one-handedness, and limitations in strength, flexibility, endurance, and mobility.[5, 6]

Yet many persons with upper limb amputation abandon or reject their prostheses because they are not satisfied with available prosthetic choices.[7, 8] Studies show that rates of prosthetic rejection vary for different types of prostheses, with rejection of myoelectric hands, body-powered hooks and passive hands, at 39%, 50%, and 53% respectively.[9] Transradial (TR) prosthesis users have the lowest rate of rejection (6%), followed by transhumeral (TH) users (57%), and persons with shoulder disarticulation (SD; 60%).[8]

Currently available prostheses fall short of restoration of full function, which is one reason for high rates of abandonment. Upper limb prosthesis users report that the most challenging activities include household chores (40% of users), sports (30%), hobbies (22%), activities of daily living (19%), social activities (8%), and occupational activities (6%). Upper limb amputees rank improved prosthesis function XXXX as a top design priority.[7]

Over the past decade, the Department of Veterans Affairs (VA) has focused on improving the care of Veterans with amputation. Between 20%-40% of combat amputees in U.S. conflicts in the global war on terror have sustained major upper-extremity amputation.[10, 11] Government reports have raised concerns about VA amputation care. For instance, a 2008 report found that Veterans who received their prosthetic care in the VA were less satisfied than their counterparts who received care in the private sector, suggesting a quality gap in VA care.[12] A 2011 study by the Office of the Inspector General (OIG), which focused solely on combat Veterans from Operation Enduring Freedom (OEF)/Operation Iraqi Freedom (OIF), found that only 69.6% of persons with upper limb amputation were satisfied with their prostheses, leading the OIG to call for efforts to evaluate the needs of Veterans with traumatic upper limb amputations to improve their satisfaction.

A national survey of Veterans with amputation from OEF/OIF and Vietnam reported that rates of prosthetic abandonment were actually lower for OEF/OIF combat amputees (22% overall rejection rates) as compared to 30% in the Vietnam Veteran group.[13] Newer combat Veterans with unilateral upper-limb loss were found to use nearly twice as many prostheses as those from the Vietnam group, and newer combat Veterans used more “high tech” devices, (46% myoelectric and 38% body-powered) as compared to Vietnam Veterans (22% myoelectric, 78% body-powered).[14] Despite continued dissatisfaction with devices, these data indicate that there may be greater satisfaction with prostheses amongst more recent upper limb amputees, and suggest that lower rates of abandonment may reflect improvements in technology and amputation care over time.

In 2009, the VA reorganized its amputation system of care (ASOC) and made great efforts to improve quality; [15] the full implementation of the new ASOC occurred in 2011. Additionally, the VA and Department of Defense (DoD) collaborated to develop the first evidence-based Clinical Practice Guidelines (CPGs) for the rehabilitation of persons with upper limb amputation.[16] Efforts to disseminate these CPGs (released in 2014) are currently underway system-wide. The CPGs describe care paths to improve outcomes in postoperative pain, physical health, function, psychological support and well-being, patient satisfaction, reintegration, and healthcare utilization, and should, in theory, lead to better prosthetic outcomes. These CPGs may lead to improved care and outcomes across the VA and DoD.

Given major differences in types of prosthetic devices and componentry, research is needed to understand the benefits and drawbacks of currently available devices as well as novel advanced (and expensive) technologies. Therefore, the objective of our overall study was to provide comprehensive cross-sectional and longitudinal data on function, needs, preferences, and satisfaction of Veterans with major upper limb amputation. The purposes of this manuscript are to provide descriptive summary findings and nationally representative estimates of a selection of key measures from the baseline survey for respondents with unilateral and bilateral amputation, and to compare prosthetic satisfaction, and quality of life outcomes of unilateral and bilateral amputees. These data provide prevalence estimates of Veterans with upper limb amputation as well as information about satisfaction and quality of life outcomes to inform approaches to rehabilitative care and investments in technology.

## 2.0—Methods

The study consisted of development and refinement of survey content and then administration of the survey to a national sample of Veterans at baseline and at 12-month follow-up. This manuscript reports on the cognitive interviews and pretesting for survey refinement as well as baseline data collection efforts. Future reports will address the 12-month follow-up data.

### 2.1—Survey development and content

The survey instrument was designed to assess demographics, amputation history, prosthesis use, function, quality of life, satisfaction with prosthesis and amputation care, and quality of care. It also included a risk-benefit assessment of technological advances requiring surgical intervention that was developed in conjunction with the Food and Drug Administration (reported elsewhere). Both unilateral and bilateral amputation versions of the survey were developed and tested.

Following instrument development and adaptation, a pilot study was conducted in two phases: cognitive testing to identify problematic items (Phase 1), and pretesting of the full survey (Phase 2). The cognitive interview sample (Phase 1) included 10 participants; 90% male, mean age 56 years, 30% with transradial (TR), 60% with transhumeral (TH), and 10% with shoulder level amputation (SH); 60% were prosthesis users. During the telephone-administered cognitive interviews, we identified several questions that were not understood by participants, were interpreted in multiple ways, or were redundant. These items were revised or dropped from the questionnaire. Second, we identified content areas missing in the initial version of the questionnaire that were important to respondents (e.g., training received on using a prosthesis, impairment experienced on the sound side). Third, we identified some double-barreled items requiring different types of abilities (e.g., use cell phone and take notes; peel and cut vegetables) and items requiring definitions or specific examples (e.g., heavy objects defined are those over 15 pounds; primary prosthesis is the one used most often; housework such as carrying a laundry basket). Fourth, we noted questions in which additional response options were required (e.g., neurologist, primary care doctor, and no provider were added to an item about which type of providers have been involved in your amputation care in the past 12 months). Finally, we determined that specific items were needed about primary versus secondary (spare) types of prostheses and terminal devices used, and that some questions were not relevant depending on amputation level as well as the number and types of prostheses and terminal devices used. As a result, several additional skip instructions to relevant questions based on prior responses were needed. Therefore, while the initial intention was to have both a self-administered mailed version and a telephone-administered version of the instrument, the final instrument is for telephone administration only due to the complexity of the format.

The pretest sample (Phase 2) included 13 participants; mean age 59 years, 92% male, 38% TR, 46% TH, and 15% SH; 77% were prosthesis users of whom 60% used a body-powered and 40% a myoelectric/hybrid. Based on the pretesting, we continued to refine which items should be asked based on level of amputation as well as how to ask about the number and types of prostheses and terminal devices currently used. We also added additional definitions (e.g., phantom limb, residual limb; driver rehabilitation therapist). Third, we added additional clarification for time frames of some questions. Fourth, in response to continued confusion by respondents in answering questions about difficulty with participation in particular activities, we revised the format to ask respondents about the difficulty of doing a set of activities that typically require two hands without a prosthesis first and then using a primary prosthesis and terminal device (if applicable). Then we asked respondents to think about a set of one-handed activities and asked about the level of difficulty both without and with a prosthesis (if applicable). Finally, based on the timing of the interviews, we determined that a few questions needed to be dropped so that the interview averaged 45 minutes in length. See S2 Appendix for a copy of the instrument.

### 2.2 –Survey overview

The final baseline survey was comprised of multiple items drawn from the 2008 Survey for Prosthetic Use, [17] previously validated measures, and new items developed and tested for this study. Each component of the survey is described below.

#### 2.2.1—Demographics and amputation type and etiology

The demographics section included items on age, gender, marital status, number of children, gender, race/ethnicity, and employment. The amputation section included items asking about: the side and level of amputation; date, etiology of amputation; surgical history related to the amputation; and hand dominance. When the gender item was not answered at the time of interview, we utilized the gender variable available in the VA Corporate Data Warehouse (CDW).

#### 2.2.2—Prosthesis use

Respondents who reported that they were current prosthesis users were asked to identify their primary device and terminal devices, and if they used more than one type of device or terminal device to indicate which one they considered their secondary or spare device. They were then asked how these prostheses were suspended to their body.

Respondents were asked whether they had ever stopped using a prosthesis, and if abandoned, what type of device(s) they had stopped using, and all reasons for abandonment. Those who were current prosthesis users were asked to report the frequency of prosthesis use, and hours per day of use. Respondents were also asked if they had received prosthetic training, and if so, the number of visits of training, the person who provided the training, and the expertise of the person providing the training.

Additional survey sections asked about the use of prosthesis during daily activities. Finally, items about the frequency of device repairs and the frequency of visits to a prosthetist for adjustments to the socket in the past 12 months were included. Results for these items will be reported elsewhere.

#### 2.2.3—Satisfaction with the prosthesis

Prosthetic satisfaction was addressed using the Trinity Amputation and Prosthetic Experience Scale (TAPES) satisfaction scale, the OPUS Client satisfaction with devices (CSD) scale, as well as items drawn from earlier surveys.[17] The TAPES Satisfaction scale consists of 10 items addressing color, shape, noise, appearance, weight, usefulness, reliability, fit, comfort and overall satisfaction.[18] Items are rated on a 5-point scale (1 = very dissatisfied, 5 = very satisfied). Cronbach alpha for this sample was 0.88. The OPUS CSD contains 11 items relating to prosthesis weight, ease of donning, durability, fit, appearance, comfort, wear and tear from clothes, pain of wearing, skin abrasions, cost of maintenance and cost of repair. Items were rated on a 4-point scale (1 = strongly agree, 4 = strongly disagree). Nine of the items are summed to achieve the final recorded score. The two items related to cost are scored separately. Cronbach alpha for the nine-item scale in this sample was 0.81. The total CSD score was calculated by summing the CSD items. The percentile value of the CSD score (as compared to provisional normative values) was estimated for those without missing values on any items by summing the total of all items and cross-walking to the norm-based values shown on the OPUS Scoring Guide.[19] The survey also included investigator-generated items asking about desire to change devices, inability to wear the prosthesis because of poor socket fit, satisfaction with the way the prostheses and terminal device moves, and unintended movement of the prosthesis.

#### 2.2.4—Function and quality of life

The survey included validated scales and additional items related to function and quality of life. Perceived disability was measured using the 11-item QuickDASH,[20] that assesses difficulty performing activities, amount of limitation, or the extent of interference with activities as well as extent of arm, shoulder and hand pain and tingling. [21, 22] The Cronbach alpha for the QuickDASH in this sample was 0.87. Additional items asked respondents to rate the difficulty of performing 5 common activities (3 two-handed activities, and 2 one-handed activities) with and without using their primary prosthesis and terminal device.

Health Related Quality of Life was assessed using the VR-12 item, a Veteran version of the SF-12 Health Survey that produces the Physical Component Summary (PCS) (Cronbach alpha of 0.86 in this sample) and the Mental Component Summary (MCS) (Cronbach alpha of 0.88 in this sample) scores.[23, 24] Participants were also asked other investigator-generated items including whether or not they needed help from another person to perform daily activities, and if so. how many hours of help they required in a typical day.

The questionnaire included items asking about the presence of pain in the prior 4 weeks in the phantom limb, residual limb, contralateral limb, neck, and back. These items asked about the frequency of each type of pain, and the intensity for those experiencing pain. The questionnaire also included items asking respondents whether they had ever been diagnosed with any of 9 common musculoskeletal conditions in the sound side (e.g. tendinitis of wrist, elbow, finger, thumb, and rotator cuff, carpal tunnel syndrome, and arthritis), residual limb health, and pain. These items were adapted from the Reiber survey.[17] We calculated the proportion of respondents who reported any contralateral limb condition. A detailed analysis of pain and musculoskeletal conditions will be reported in future papers.

Additional items, drawn from prior surveys (results of which will be reported in future papers), pertained to difficulty with activities and participation, and were assessed using items about eating, meal preparation, housework, home maintenance, computer use, lifting and carrying. Our questionnaire also included a single item on the extent of bother from residual limb sweating in the socket, drawn from the residual limb health subscale of the Prosthetics Evaluation Questionnaire.[25] Other items asked about body image, flashback/nightmares related to the amputation, difficulty concentrating, sense of embodiment of the prosthesis, and confidence using the prosthesis.

#### 2.2.5—Amputation care

The questionnaire included a section on amputation related care and care quality. Investigator-generated items asked about where the respondent had ever gone for amputation-related care, and those who indicated that they had ever gone to a VA Amputation clinic or Department of Defense Amputation clinic were asked the year of most recent visit.

The questionnaire also included the OPUS Client Satisfaction with Services scale (CSS).[26] Respondents were asked a series of investigator generated questions that addressed aspects of clinical practice guidelines for rehabilitation of persons with upper limb amputation. The CSS and the investigator generated items will be described and reported upon in a separate manuscript addressing quality of care.

#### 2.2.6—Risk benefit assessment and technology acceptance

The questionnaire also included a section on risk benefit and technology acceptance of potentially new prosthetic devices, capabilities, and suspension methods. Findings related to these items will be reported in separate manuscripts.

### 2.3—Survey recruitment and data collection

Our goal was to include a representative sample of Veterans with major upper limb amputation who received care in the VA between 2010–2016, defined in our study as amputation at the forequarter, shoulder disarticulation, transhumeral (TH), elbow disarticulation, transradial (TR), or wrist disarticulation level, The sampling frame was identified from VA CDW sources including Inpatient, Outpatient, and Fee domains; the main source for information regarding VHA Benefit compensation and pension benefits paid to veterans and their beneficiaries; and Veteran’s Benefits Administration (VBA) disability ratings. A list of diagnosis and procedure codes used to identify the sample is provided in S1 Appendix.

All non-deceased Veterans with valid addresses and phone numbers were sent an initial recruitment package containing an invitation letter, a study information sheet explaining the study, and a card with stamped envelope to return if they wished to opt out of participation. Veterans who did not opt-out of study participation by returning the postcard or calling the study telephone number within 30 days were contacted by the study interviewers. To maximize study recruitment, up to ten attempts were made to contact potential participants. All participants provided oral informed consent to participate. A waiver of documentation of informed consent was obtained from the VA Central IRB. All surveys were conducted via telephone by trained interviewers and were approximately 45 minutes in length. Separate versions of the survey were administered to unilateral and bilateral amputees. The bilateral version included all questions in the unilateral version but asked collected information on key variables (e.g. amputation etiology, prosthesis use) for both the left and right sides.

### 2.4—Statistical methods

Response (RR) and cooperation (CR) rates were calculated using American Association of Public Opinion Research guidelines (AAPOR RR4 and CR4).[27] In RR4 and CR4 those with partial interviews are considered as completers. The denominator of eligible subjects in the RR4 includes an estimate for the proportion of cases of unknown eligibly that are actually eligible. The cooperation rate does not include those who could not be reached for the screener or survey. Using data available in CDW we compared age, gender and year of last encounter at the VA of survey responders and non-responders to assess potential bias in survey respondents. In 3 cases where CDW data differed from self-reported gender, we used the self-report data to categorize gender.

We conducted descriptive analyses to characterize the groups of respondents with unilateral and bilateral amputation. We compared scores for prosthetic satisfaction (TAPES, CSD) for unilateral and bilateral amputees using t-tests and non-parametric Wilcoxon rank sum (WRS) tests. We also compared quality of life outcomes including the QuickDASH, VR-12 PCS and MCS using t-tests for unilateral versus bilateral amputation. We conducted post-hoc analyses to estimate the magnitude of effect size that we were powered to detect.

## 3.0—Results

### 3.1- Sampling frame and response rate

**Fig 1:**
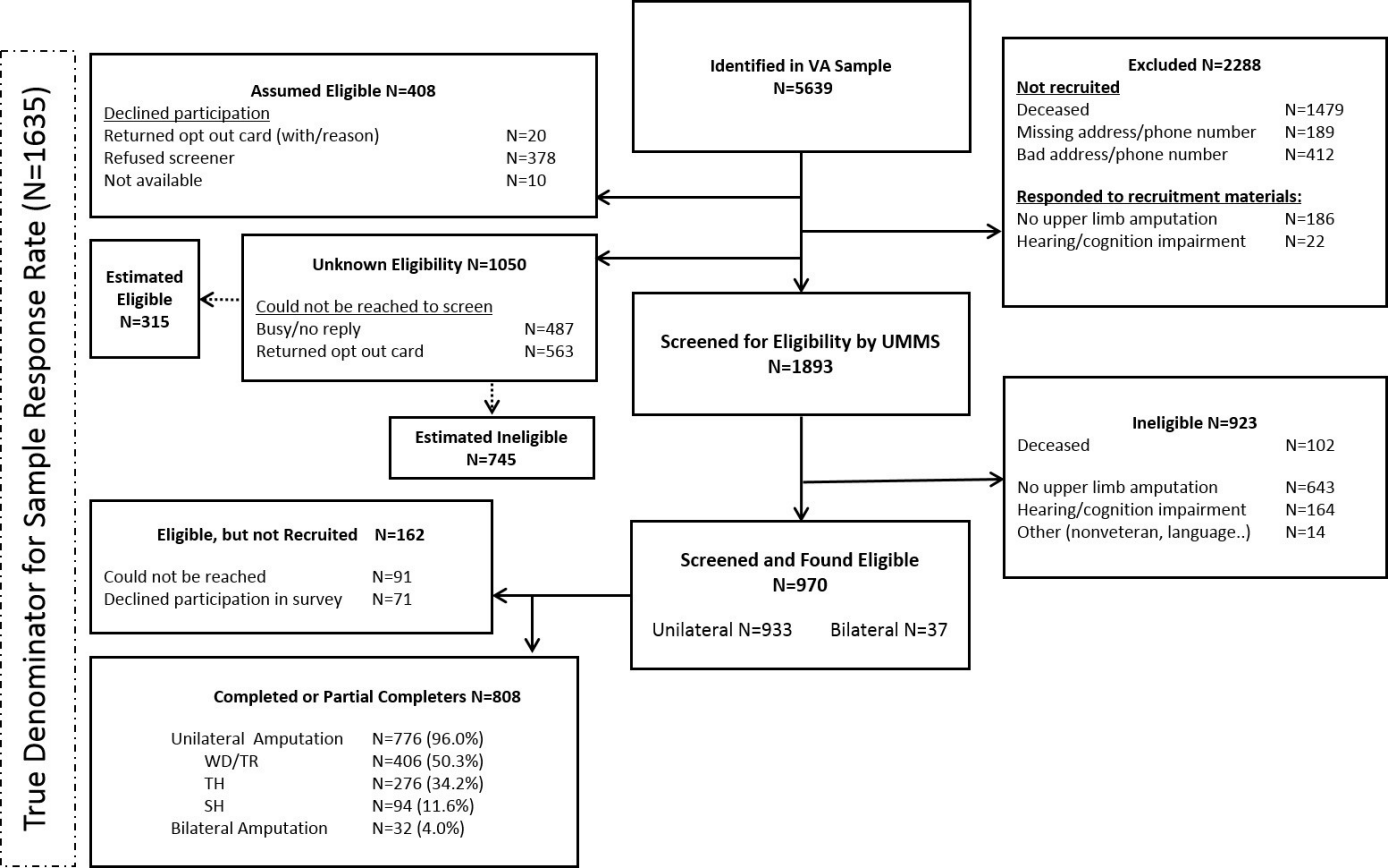
Flow diagram.

We identified 5639 persons (shown in Fig 1) with a diagnosis of upper limb amputation who had been seen at the VA between 2011 and 2015. We excluded 2080 persons, 1479 of whom were found to be deceased, and 601 who were missing valid addresses and phone numbers. Recruitment materials with opt out cards were sent to the remaining 3559 persons. Two hundred eight persons who responded to the recruitment invitation told us that they did not meet study eligibility criteria. Four hundred eight persons declined participation, and 1050 could not be reached for screening. We screened 1893 persons, 923 were found to be ineligible and 970 found to be eligible. Eight hundred eight (83%) of those screened to be eligible were recruited into the study. The final response rate (RR) and cooperation rate (CR) was 47.7% and 63.3%, respectively [27].

Table 1 compares the 808 survey respondents and the 1620 eligible persons who did not respond. On average, responders were 1.8 years younger (p = 0.0059), more often female (p = 0.0289), and had a more recent year of VA utilization (p = 0.0109).

**Table 1:** Comparison of survey respondents and non-respondents.

	Not Recruited (Eligible or Unknown Eligibility) (N = 1620)	Completers (N = 808)	
	Mean (SD)	Mean (SD)	T-test p
Age (years)	65.0 (16.0)	63.2 (14.2)	**0.0059**
	**N (%)**	**N (%)**	**Chisq p**
Gender			**0.0289**
Female	22 (1.4)	21 (2.6)	
Male	1598 (98.6)	787 (97.4)	
Race			0.3211
White	1106 (68.2)	558 (69.1)	
Black	202 (12.5)	82 (10.2)	
Other/Mixed	63 (3.98)	30 (3.7)	
Unknown	250 (15.4)	138 (17.1)	
Last year of VA visit*			**0.0109**
2010	5 (0.3)	1 (0.1)	
2011	15 (0.9)	5 (0.6)	
2012	19 (1.2)	2 (0.3)	
2013	36 (2.2)	7 (0.9)	
2014	30 (1.9)	14 (1.7)	
2015	63 (3.9)	20 (2.5)	
2016	1452 (89.6)	759 (93.9)	

*last year of visit between 1/1/2010-12/31/2016

### 3.2—Sample characteristics

Seven hundred eighty-eight persons completed the survey in its entirety, while 20 persons completed at least part of the survey. Table 2 compares demographic data for the sample of 776 unilateral amputees and 32 bilateral amputees. Mean age was 63.3 (sd 14.1), and 787 (97.5%) were male. Seventy-five percent of the sample classified themselves as white, and 8.6% identified themselves as Hispanic or Latino. Only 13% of the sample reported that they were currently working full- or part- time, while 70% were retired. On average, these amputees had lost their limbs 31.4 (sd 18.3) years prior. Among unilateral amputees, the largest amputation level group was transradial (36.1%), followed by above the elbow (30.4%), at the wrist joint (16.2%), at the shoulder (9.2%), at the elbow (5.2%) and forequarter (3.0%). The most common etiologies of amputation (respondents indicated all etiologies that applied) were accident (62.1% unilateral, 62.5% bilateral), “other” (54% unilateral, and 65.6% left side-71.9% right-side bilateral), and combat injury (35.5% unilateral, 28.1% bilateral). Burns were listed as a prevalent cause of amputation for bilateral amputees (40.6% left and right combined).

**Table 2:** Demographics characteristics of unilateral and bilateral amputee respondents.

	Unilateral Amputees N = 776	Bilateral Amputees N = 32		All N = 808
	Mean (sd)	Mean (sd)		Mean (sd)
**Age (Years)**	63.2 (14.1)	63.6 (15.3)		63.3 (14.1)
Missing (n)	24	0		24
**Years since initial amputation (either side)**	31.3 (18.4)	32.8 (18.1)		31.4 (18.3)
**Years since amputation (second side)**	31.3 (18.4)	32.6 (18.3)		31.4 (18.3)
Missing (n)	21	0		21
	**n (%)**	**n (%)**		**n (%)**
**Year of initial amputation**				
1940–1949	6 (0.8)	1 (3.1)		7 (0.9)
1950–1959	20 (2.7)	2 (6.3)		22 (2.8)
1960–1969	182 (24.1)	4 (12.5)		186 (23.6)
1970–1979	136 (18.0)	6 (18.8)		142 (18.0)
1980–1989	83 (11.0)	6 (18.8)		89 (11.3)
1990–1999	76 (10.1)	5 (15.6)		81 (10.3)
2000–2003	44 (5.8)	2 (6.3)		46 (5.8)
2004–2006	51 (6.8)	1 (3.1)		52 (6.6)
2007–2009	46 (6.1)	1 (3.1)		47 (6.0)
2010–2013	84 (11.1)	4 (12.5)		88 (11.2)
2014–2016	27 (3.6)	0 (0.0)		27 (3.4)
Missing (n)	21	0		21
**Gender**				
Male	755 (97.3)	32 (100.0)		787 (97.5)
Female	21 (2.7)	0 (0.0)		21 (2.6)
Missing (n)	24	0		24
**Race**				
White	583 (77.5)	22 (68.8)		605 (74.9)
Black	86 (11.4)	3 (9.4)		89 (11.0)
Native American	5 (0.7)	0 (0.0)		5 (0.6)
Other (including mixed race)	30 (4.0)	4 (12.5)		34 (4.2)
Unknown	48 (6.4)	3 (9.4)		75 (9.3)
Missing (n)	24	0		24
**Hispanic or Latino**				
Yes	62 (8.2)	5 (15.6)		67 (8.6)
No	678 (90.2)	26 (81.3)		704 (89.8)
Unknown	12 (1.6)	1 (3.1)		13 (1.7)
Missing (n)	24	0		24
**Employment**				
Employed full-time	73 (9.7)	1 (3.1)		74 (9.4)
Employed part-time	31 (4.1)	13 (40.6)		31 (4.0)
Student	20 (2.7)	0 (0.0)		20 (2.6)
Retired, but employed after amputation	373 (49.6)	13 (40.6)		386 (49.2)
Retired, but not employed after amputation	152 (20.2)	5 (15.6)		165 (21.1)
On medical leave	9 (1.2)	0 (0.0)		9 (1.2)
Other	93 (12.4)	0 (0.0)		98 (12.5)
Unknown	1 (0.1)	0 (0.0)		1 (0.1)
Missing (n)	24	0		24
**Laterality of amputation**				
Unilateral Right	370 (47.7)	0 (0.0)		370 (45.8)
Unilateral left	406 (52.3)	0 (0.0)		406 (50.3)
Bilateral	0 (0.0)	32 (100.)		32 (4.0)
**Amputation level**				
		Left	Right	
Forequarter	23 (3.0)	1 (3.1)	0 (0.0)	
At the shoulder joint	71 (9.2)	1 (3.1)	1 (3.1)	
Above the elbow	236 (30.4)	5 (15.6)	4 (12.5)	
At the elbow	40 (5.2)	14 (43.8)	1 (3.1)	
Below the elbow	280 (36.1)	10 (31.3)	20 (62.5)	
At the wrist joint	126 (16.2)	0 (0.0)	6 (18.8)	
Through the hand	0 (0.0)	1 (3.1)	0 (0.0)	
**Etiology of amputation (may be more than one)**				
Combat injury	275 (35.5)	9 (28.1)	9 (28.1)	
Accident	481 (62.1)	20 (62.5)	20 (62.5)	
Burn	81 (10.5)	13 (40.6)	13 (40.6)	
Cancer	30 (3.9)	0 (0.0)	0 (0.0)	
Diabetes	11 (1.4)	0 (0.0)	1 (3.1)	
Infection	86 (11.1)	9 (28.1)	8 (25.0)	
Other	417 (54.0)	21 (65.6)	23 (71.9)	
Missing (n)	3	0	0	
**Amputation of lower limb**				
Yes	94 (12.1)	8 (25.0)		
No	682 (87.9)	24 (75.0)		
**Amputation of lowerlimb**	N = 94	N = 8		
Right Side	39 (41.5)	1 (12.5)		
Left Side	23 (24.5)	1 (12.5)		
Both Sides	32 (34.0)	6 (75.0)		

### 3.3—Prosthesis use

Sixty percent of unilateral amputees said that they were prosthesis users (Table 3). Ninety-one percent of bilateral amputees used a prosthesis on at least one side. Only 6.8% of unilateral amputees had never used a prosthesis. Fifty percent of unilateral amputees reported that they had ever stopped using a prosthesis, most often a body powered device (36.4%). In contrast 34.4% of bilateral amputees reported that they had ever stopped using a prosthesis, most commonly a body-powered device (28.1%). Amongst unilateral amputees, about 40% had received their most recent prosthesis within the prior 2 years (23.6% within the prior year). However, 32.8% reported that they received their most recent device more than 4 years prior. In contrast 41% of bilateral amputees had received at least one of their devices within the past 2 years (21% within the prior year). Thirty seven percent of unilateral amputees reported that they used 2 or more prostheses, and 24% of bilateral amputees used 2 or more prostheses for at least one side. Body powered devices were the most common primary prosthesis type used (70.9% unilateral amputees, 79% bilateral left and right sides).

**Table 3:** Type of prostheses, terminal devices and suspension methods: Comparison of unilateral and bilateral amputees.

	**Unilateral Amputees** **N = 776**	**Bilateral Amputees** **N = 32**	
		**Left**	**Right**
	n (%)	n (%)	
**Currently use a prosthesis**			
Yes	461 (60.0)	25 (78.1)	27 (84.4)
No	254 (33.0)	7 (21.9)	5 (15.8)
*Never Used Prosthesis	52 (6.8)	0 (0.0)	0 (0.0)
Unknown	2 (0.3)	0 (0.0)	0 (0.0)
Missing (n)	7	0	
**Have you ever stopped using a prosthesis?**			
Yes	379 (49.9)	11 (34.4)	
No	327 (43.1)	21 (65.6)	
*Never Used Prosthesis	52 (6.9)	0 (0.0)	
Unknown	1 (0.1)	0 (0.0)	
Missing (n)	17	0	
**Were any of the prostheses that you stopped using?**			
Body powered	276 (36.4)	9 (28.1)	
Myoelectric	135 (17.8)	3 (9.4)	
Hybrid	26 (3.4)	0 (0.0)	
*Never Used Prosthesis	52 (6.9)	0 (0.0)	
*Never stopped using ANY prosthesis	327 (43.1)	21 (65.6)	
Unknown	21 (2.8)	0 (0.0)	
Missing (n)	18	0	
**Prosthesis users**	**Unilateral** N = 461	**Left** **N = 25**	**Right** **N = 27**
**Prosthesis users: most recent prosthesis received**			
< 3 months	29 (6.3)	2 (8.0)	4 (14.8)
3–6 months	42 (9.1)	2 (8.0)	3 (11.1)
6–12 months	38 (8.2)	4 (16.0)	4 (14.8)
12–24 months	90 (19.5)	6 (24.0)	6 (22.2)
2–4 years	109 (23.6)	6 (24.0)	6 (22.2)
4 + years	151 (32.8)	5 (20.0)	4 (14.8)
Unknown	2 (0.4)	0 (0.0)	0 (0.0)
**Number of prostheses used**			
One	291 (63.1)	21 (84.0)	20 (74.1)
Two or more	170 (36.9)	4 (16.0)	7 (25.9)
**Primary type of prosthesis used**			
Body powered	326 (70.9)	19 (76.0)	21 (77.8)
Myoelectric	96 (20.9)	3 (12.0)	4 (14.8)
Hybrid	6 (1.3)	0 (0.0)	0 (0.0)
Cosmetic	22 (4.8)	3 (12.0)	2 (7.4)
Sports/recreation	6 (1.3)	0 (0.0)	0 (0.0)
Unknown	4 (0.9)	0 (0.0)	0 (0.0)
Missing (n)	1	0	0
**Suspension type for primary prosthesis**			
Suction	156 (34.0)	5 (20.0)	8 (29.6)
Gel or silicone liner with pin	91 (19.8)	2 (8.0)	2 (7.4)
Vacuum	51 (11.1)	2 (9.0)	5 (18.5)
Self-suspending because of the socket shape	172 (37.5)	11 (44.0)	11 (40.7)
Harnessing	345 (75.2)	21 (84.0)	22 (81.5)
External strap	62 (13.5)	4 (16.0)	3 (11.1)
Unsure	1 (0.2)	0 (0.0)	0 (0.0)
Missing (n)	2	0	0
**Number of terminal devices used**			
One	259 (56.4)	23 (92.0)	23 (85.2)
Two or more	195 (42.5)	2 (8.0)	4 (14.8)
Unknown	5 (1.1)	0 (0.0)	0 (0.0)
Missing (n)	2	0	0
**Users of one or more terminal devices**	**Unilateral** **N = 454**	**Left** **N = 25**	**Right** **N = 27**
**Primary type of terminal device used**			
Body-powered hook	281 (62.2)	18 (72.0)	20 (74.1)
Greiffer	6 (1.3)	0 (0.0)	0 (0.0)
Power hook (ETD)	17 (3.8)	0 (0.0)	4 (14.8)
Sensor Speed Hand	11 (2.4)	2 (8.0)	0 (0.0)
I-Limb	14 (3.1)	0 (0.0)	0 (0.0)
Michaelangelo hand	7 (1.6)	0 (0.0)	0 (0.0)
Bebionic hand	28 (6.2)	0 (0.0)	0 (0.0)
Other	77 (17.0)	5 (20.0)	3 (11.1)
Unknown	11 (2.4)	0 (0.0)	0 (0.0)
Missing (n)	4	0	0
**Prosthetic users with two or more prostheses**	**Unilateral** **N = 170**	**Left** **N = 4**	**Right** **N = 7**
**Secondary type of prosthesis used**			
Body powered	74 (43.8)	2 (50.0)	3 (42.9)
Myoelectric	63 (37.3)	2 (50.0)	3 (42.9)
Hybrid	3 (1.8)	0 (0.0)	0 (0.0)
Cosmetic	5 (3.0)	0 (0.0)	1 (14.3)
Sports/recreation	20 (11.8)	0 (0.0)	0 (0.0)
Unknown	4 (2.4)	0 (0.0)	0 (0.0)
Missing (n)	1	0	0

Forty-three percent of unilateral amputees reported that they used two or more types of terminal devices, as compared to about 14% of bilateral amputees who used two or more terminal devices on at least one side. The most common types of primary terminal devices were body powered hooks (unilateral: 62%, bilateral: 72% left, 74.0% right. Multi-degree of freedom terminal devices (including the I-limb, Michaelangelo Hand and Bebionic) were used by 10.8% of unilateral amputees and 0% of bilateral amputees. The most prevalent suspension methods were self-suspending (75.2% unilateral, 84.0% left and 81.5% right bilateral), followed by gel or silicone liners with pin (37.5% unilateral, 44.0% left and 40.7% right bilateral), and suction (34.0% unilateral, 20% left and 29.6% right bilateral).

**Fig 2:**
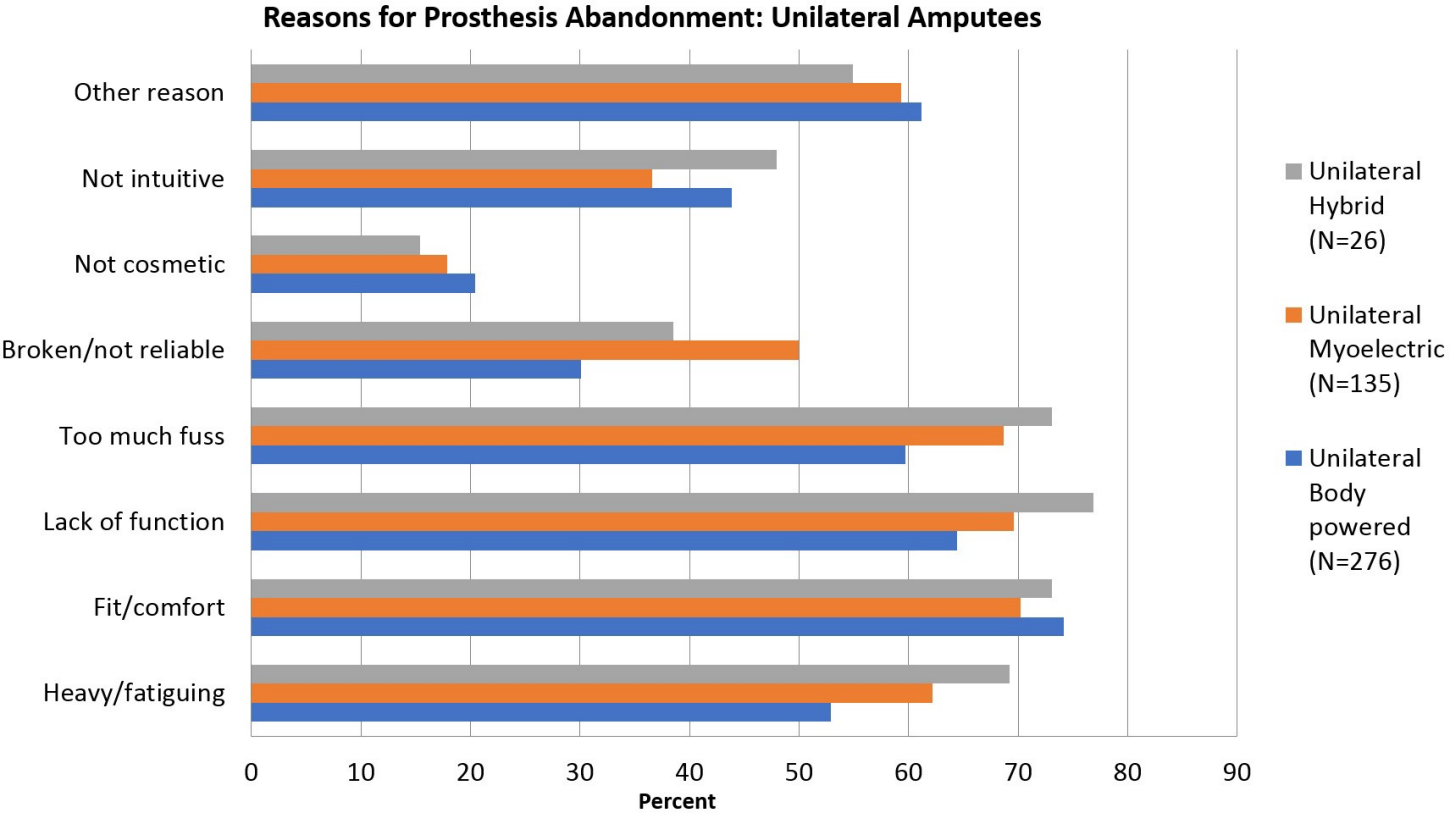
Reasons for prosthesis abandonment by type of device: Unilateral amputees.

**Fig 3:**
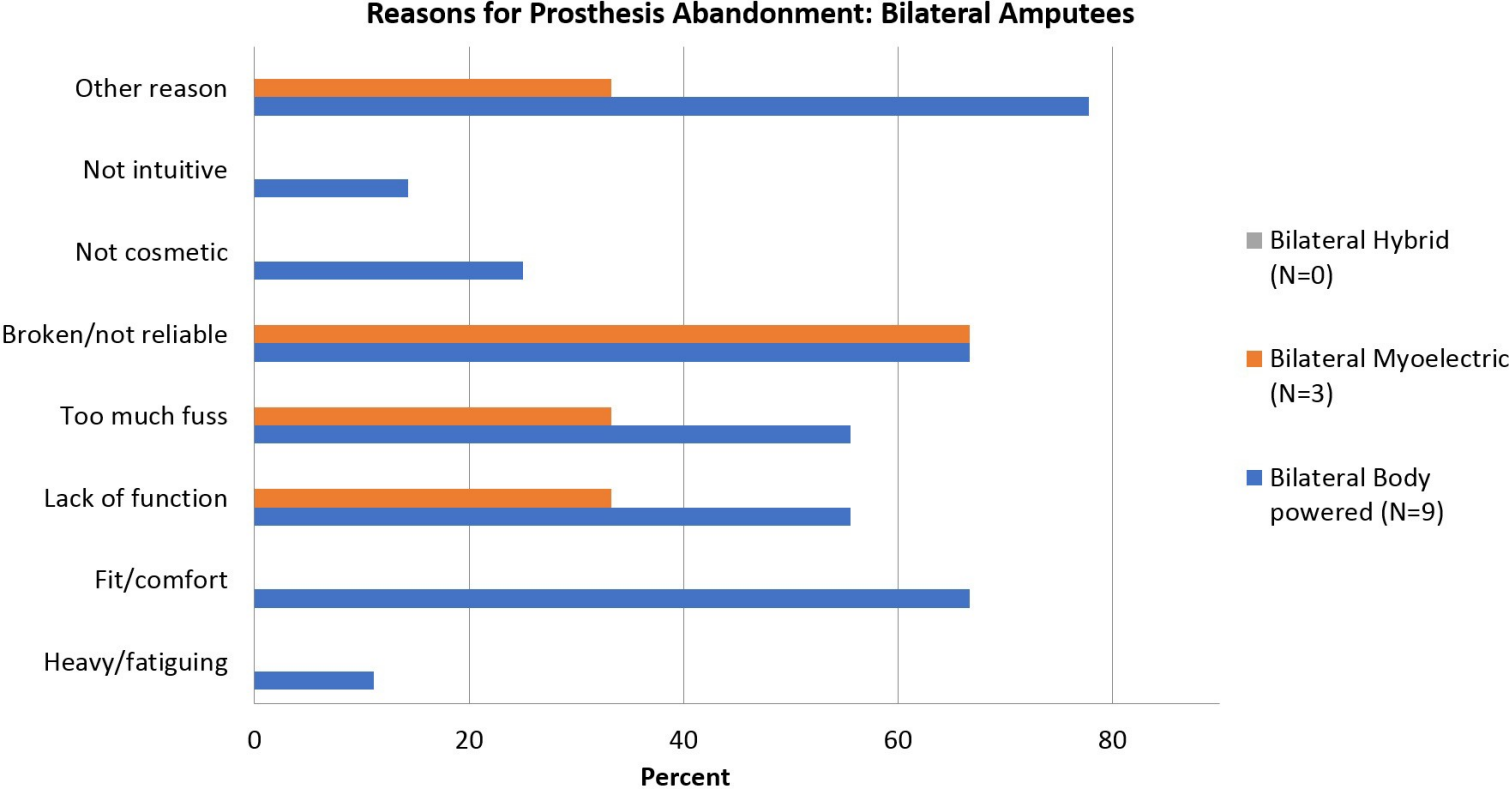
Reasons for prosthesis abandonment by type of device: Bilateral amputees.

The reasons reported for abandoning each type of device is shown in Fig 2. For unilateral amputees, the most common reasons for abandoning all types of devices were lack of function, too much fuss, fit/comfort and heaviness/fatigue. There were differences in reasons for abandonment by prosthesis type, for example, 50.0% of myoelectric abandoners reported that the device was broken or unreliable, as compared to 38.0% of hybrid abandoners and 30.1% of body powered abandoners. A greater proportion of myoelectric and hybrid device abandoners cited too much fuss, lack of function, and heavy/fatiguing as compared to body powered users. For the 11 bilateral amputees who abandoned a device (Fig 3), the most common reasons were broken/unreliable devices, fit/comfort, and other reasons. Body-powered users listed too much fuss, lack of function, and other reasons more often than myoelectric users.,.

**Fig 4:**
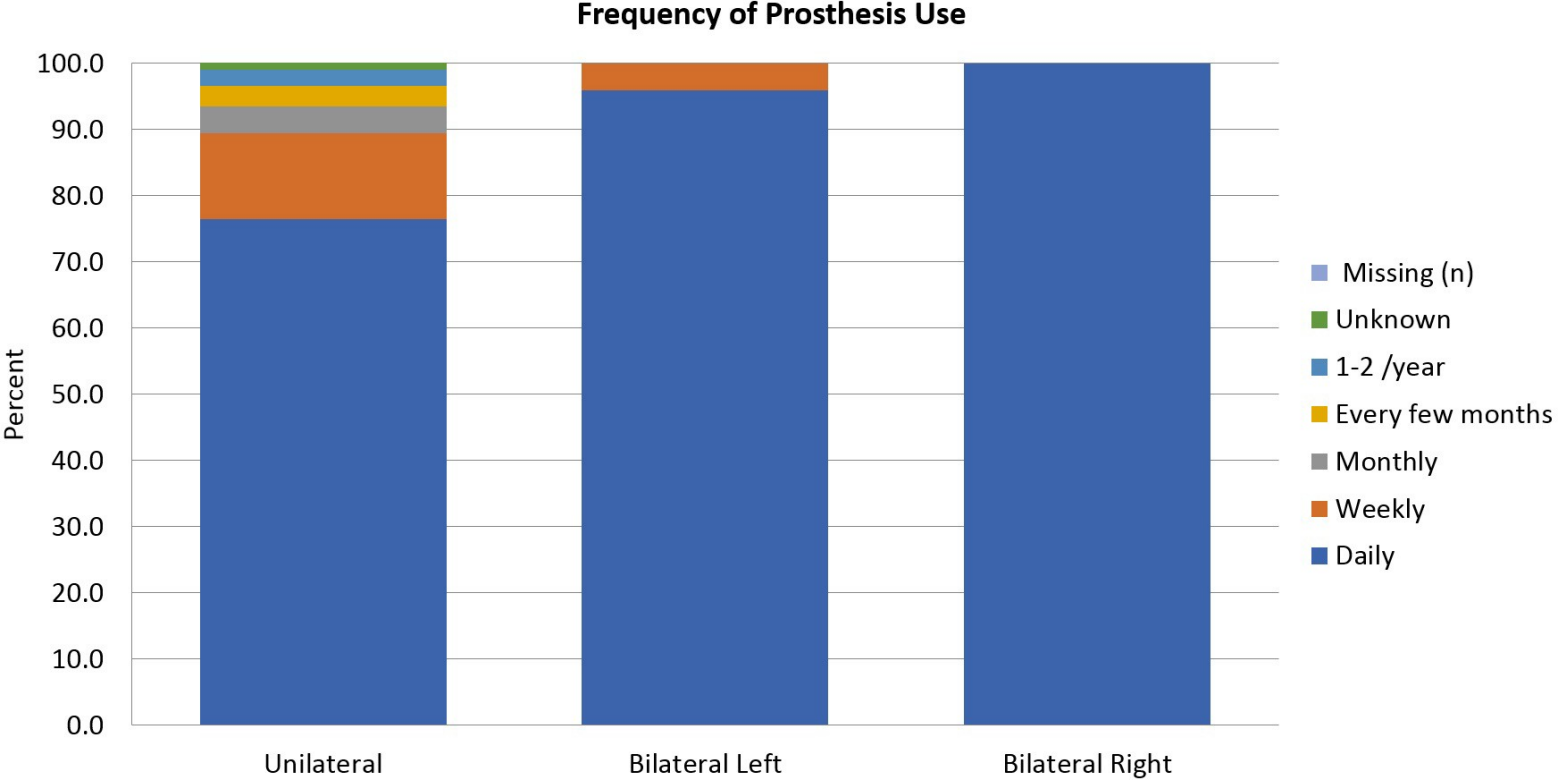
Frequency of prosthesis use.

**Fig 5:**
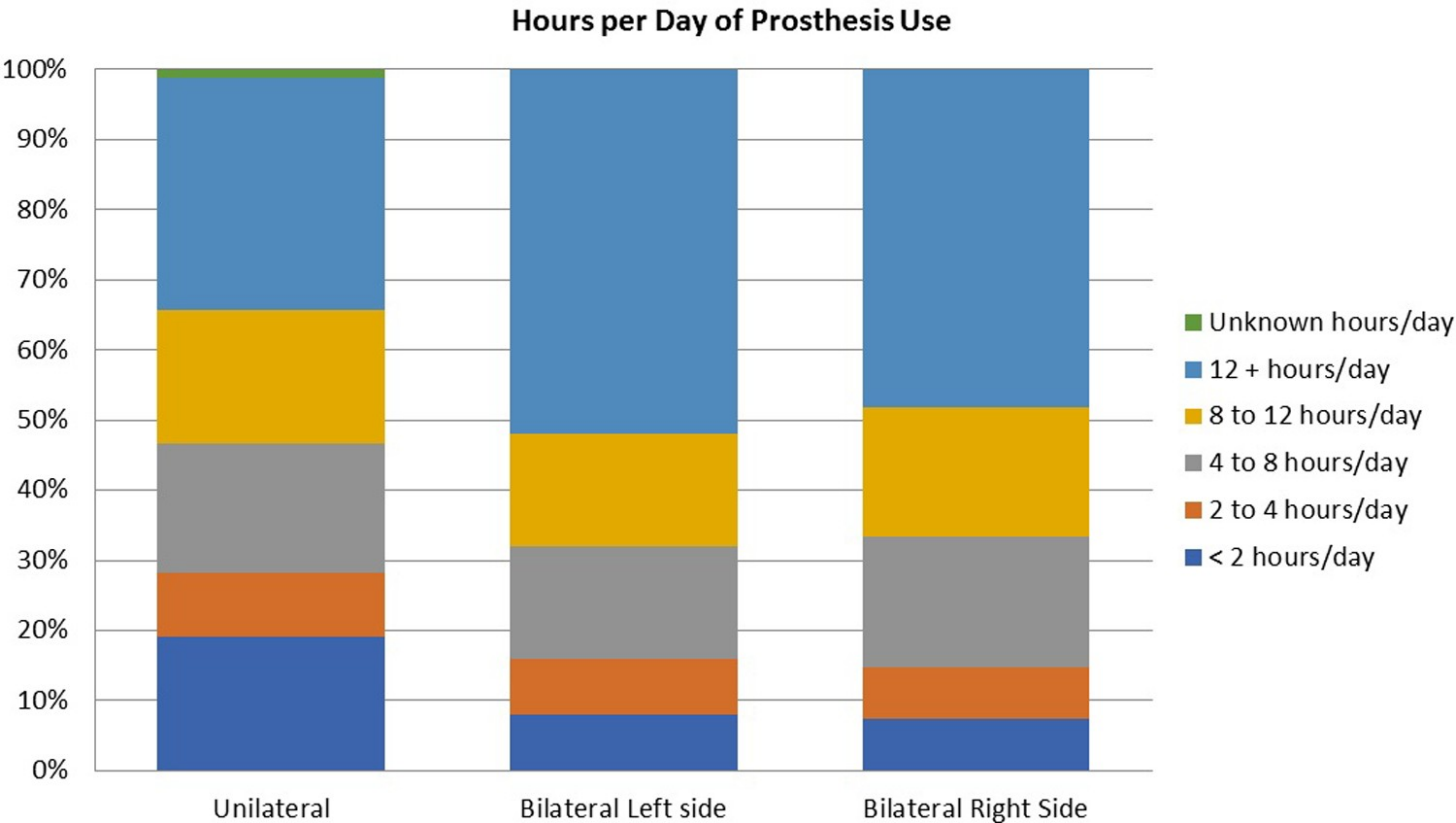
Hours of prosthesis use per day.

Hours of use of the prosthesis are compared graphically in Fig 4. Seventy seven percent of unilateral amputees used their devices daily, and 52% reported that they used their devices 8 or more hours per day. (Fig 5). Nineteen percent used their devices less than 2 hours per day. One hundred percent of bilateral amputees used at least one prosthesis daily, and 76% used at least one of their prostheses 8 hours per day or more, while about 7% used at least one less than 2 hours per day. (Fig 5)

### 3.4—Prosthesis training

Seventy one percent of unilateral amputees and 59% of bilateral amputees had received training to use their first prosthesis. A slightly lower proportion (66% unilateral, 48.0% left and 55.6% right bilateral) had received training to use their current primary prosthesis. The distribution of training visits is shown in Table 4. Overall, 28% of unilateral amputees received 1–3 training visits. Twenty one percent of bilateral amputees had received 1–3 training visits for the prosthesis they used on their left side, and 14.8% received this amount of training for the prosthesis that they used on their right side. At the other extreme, 14.8% of unilateral amputees received more than 30 training visits. Seventeen percent of bilateral amputees received more than 30 hours of training for their left side, and 30% received it for their right side. Prosthetic training visits were conducted most often by a PT or OT (54.5% unilateral, 41.7% for bilateral left and 60.0% for bilateral right), and less frequently by a prosthetist (unilateral: 40.9%; bilateral 33.3%on left, 26.7% on right). Respondents rated the skill level of their trainers high, with only 2% of unilateral and 0% of bilateral amputees stating that their trainers were “not at all skilled.”

**Table 4:** Prosthesis training.

	**Unilateral Amputees** **N = 776**	**Bilateral Amputees** **N = 32**	
	**N (%)**	**N (%)**	**N (%)**
**Received training to use first prosthesis**			
Yes	545 (70.9)	19 (59.4)	
No	165 (21.5)	13 (40.6)	
Never Used Prosthesis	52 (6.8)	0 (0.0)	
Unknown	7 (0.91)	0 (0.0)	
Missing (n)	7	0	
**Prosthesis users**	**Unilateral N = 461**	**Left** **N = 25**	**Right** **N = 27**
**Received training to use current prosthesis**			
Yes	301 (66.0)	12 (48.0)	15 (55.6)
No	153 (33.6)	12 (48.0)	12 (44.4)
Unknown	2 (0.4)	1 (4.0)	0 (0.0)
Missing (n)	5	0	0
**Number of training visits**			
1 to 3	127 (28.0)	5 (20.8)	4 (14.8)
4 to 10	53 (11.7)	1 (4.2)	2 (7.4)
11 to 20	22 (4.9)	1 (4.2)	1 (3.7)
21 to 30	21 (4.6)	0 (0.0)	0 (0.0)
More than 30	67 (14.8)	4 (16.7)	8 (29.6)
No Training	153 (33.7)	12 (50.0)	12 (44.4)
Unknown	11 (2.4)	1 (4.2)	0 (0.0)
Missing (n)	7	0	0
**Received training**	**Unilateral N = 301**	**Left** **N = 12**	**Right** **N = 15**
**Who conducted prosthetic training**			
Prosthetist	123 (40.9)	4 (33.3)	4 (26.7)
PT/OT	164 (54.5)	5 (41.7)	9 (60.0)
Other	6 (2.0)	3 (25.0)	2 (13.3)
Unknown	8 (2.7)	0 (0.0)	0 (0.0)
**Rating of trainer skill level**			
Not at all skilled	6 (2.0)	0 (0.0)	0 (0.0)
Adequately skilled	69 (22.9)	3 (25.0)	4 (26.7)
Highly skilled	217 (72.1)	9 (75.0)	10 (66.7)
Unknown	9 (3.0)	0 (0.0)	1 (6.7)

### 3.5—Satisfaction with the prosthesis, health function and quality of life

Prosthetic satisfaction ratings are shown in Table 5. The overall TAPES scores

indicated that both unilateral and bilateral amputees were somewhat satisfied with their prostheses: unilateral amputees mean scores 3.9 (0.6), bilateral mean scores 3.8 (0.7).

**Table 5:** Satisfaction with primary prosthesis (prosthesis users only).

		Unilateral N = 461		Bilateral ^ N = 29	t test	WRS+
Prosthesis users only		Mean (sd)		Mean (sd)	p-value	p-value
**TAPES satisfaction scale#**						
Color	449	4.0 (0.8)	28	4.0 (0.6)	0.8094	0.7292
Shape	449	4.0 (0.8)	29	4.1 (0.6)	0.5243	0.6541
Noise	430	4.0 (0.8)	28	3.9 (1.0)	0.2594	0.4042
Appearance	450	3.9 (0.9)	29	3.8 (0.9)	0.7412	0.6473
Weight	453	3.8 (1.0)	29	3.7 (1.1)	0.7128	0.7608
Usefulness	450	3.8 (1.1)	29	3.9 (1.0)	0.7302	0.7342
Reliability	449	3.9 (1.0)	29	3.8 (1.0)	0.6469	0.5302
Fit	449	3.9 (1.0)	29	3.8 (1.0)	0.4279	0.3013
Comfort	450	3.6 (1.1)	29	3.5 (1.1)	0.5423	0.4997
Overall Satisfaction	447	4.0 (0.9)	29	3.9 (0.8)	0.8240	0.6416
Average total TAPES satisfaction score	453	3.9 (0.6)	29	3.8 (0.7)	0.6654	0.5858
**OPUS Client satisfaction with devices (CSD)** *						
My prosthesis fits well	448	1.9 (0.8)	29	2.1 (0.8)	0.2709	0.2171
The weight of my prosthesis is manageable	451	1.8 (0.6)	29	1.8 (0.4)	0.7660	0.4701
My prosthesis is comfortable throughout the day	448	2.2 (0.8)	28	2.1 (0.5)	0.4699	0.5055
It is easy to put on my prosthesis	450	1.8 (0.7)	29	1.9 (0.5)	0.5586	0.3979
My prosthesis looks good	443	2.0 (0.7)	27	2.0 (0.6)	0.8022	0.5823
My prosthesis is durable	447	1.9 (0.7)	29	2.1 (0.7)	0.6820	0.1106
My clothes are free of wear and tear from my prosthesis	449	2.8 (0.9)	29	3.1 (0.8)	0.0547	0.0760
My skin is free of abrasions and irritations	448	2.3 (0.8)	29	2.3 (0.7)	0.5921	0.6293
My prosthesis is pain-free to wear	444	2.2 (0.8)	28	2.3 (0.6)	0.9401	0.9803
I can afford out-of-pocket expenses to purchase and maintain prosthesis	385	3.0 (0.9)	24	3.0 (0.9)	0.7722	0.7881
I can afford to repair or replace my prosthesis as soon as needed	386	2.9 (0.9)	23	3.2 (0.8)	0.2140	0.2578
OPUS CSD total score	347	25.0 (5.1)	20	25.7 (4.5)	0.5694	0.6412
OPUS CSD crosswalk estimated percentile score	347	49.6 (10.2)	20	51.2 (7.7)	0.4871	0.6412
**Additional satisfaction related items**						0.5123
My terminal device is appropriately sized for me	318	1.8 (0.6)	22	1.9 (0.4)	0.4626	0.2994
Overall, my prosthetic device is appropriately sized	318	1.8 (0.6)	22	2.0 (0.5)	0.1362	0.0987
I am self-conscious about wearing my prosthesis	447	2.9 (0.9)	29	2.9 (0.8)	0.8906	0.8983
Desire to change devices	435	2.7 (0.9)	28	2.8 (0.8)	0.4553	0.5123
Inability to wear the prosthesis due to fit	446	3.1 (0.8)	28	3.1 (0.6)	0.9362	0.6520
Satisfaction with prosthesis/terminal device movement	452	1.8 (0.8)	29	1.9 (0.7)	0.9074	0.9276
Unintended movement	447	2.5 (0.9)	29	2.4 (0.8)	0.4878	0.4861

^Satisfaction with dominant side

#Response categories for TAPES: 1 = very dissatisfied, 2 = dissatisfied, 3 = neither satisfied nor dissatisfied, 4 = satisfied, 5 = very satisfied

*Response categories for CSD and additional satisfaction related items: * 1 = Strongly Agree, 2 = Agree, 3 = Disagree 4 = Strongly Disagree

+WSR = Wilcoxon rank sum test.

Comfort was the lowest rated individual item, but was still in the neither satisfied nor dissatisfied range. The total CSD scores were 25.0 (5.1) and 25.7 (4.5) for unilateral and bilateral amputees, respectively. The cross-walked scores were 49.6 (10.2) for unilateral and 51.2 (7.7) for bilateral amputees. The items rated most highly pertained to fit and durability of the prosthesis. The items rated most poorly pertained to self-consciousness, clothing wear and tear, and device costs. The only differences between unilateral and bilateral amputees related to CSD items was in the item “my clothes are free from wear and tear,” bilateral amputees disagreed more with this statement, however the difference was small and did not reach statistical significance (P = 0.054). In general, participants did not want to change their prosthesis to another type, could wear their prosthesis because of fit, were satisfied with prosthesis/terminal device movement but indicated that their prosthesis sometimes moved in unintended ways.

**Fig 6:**
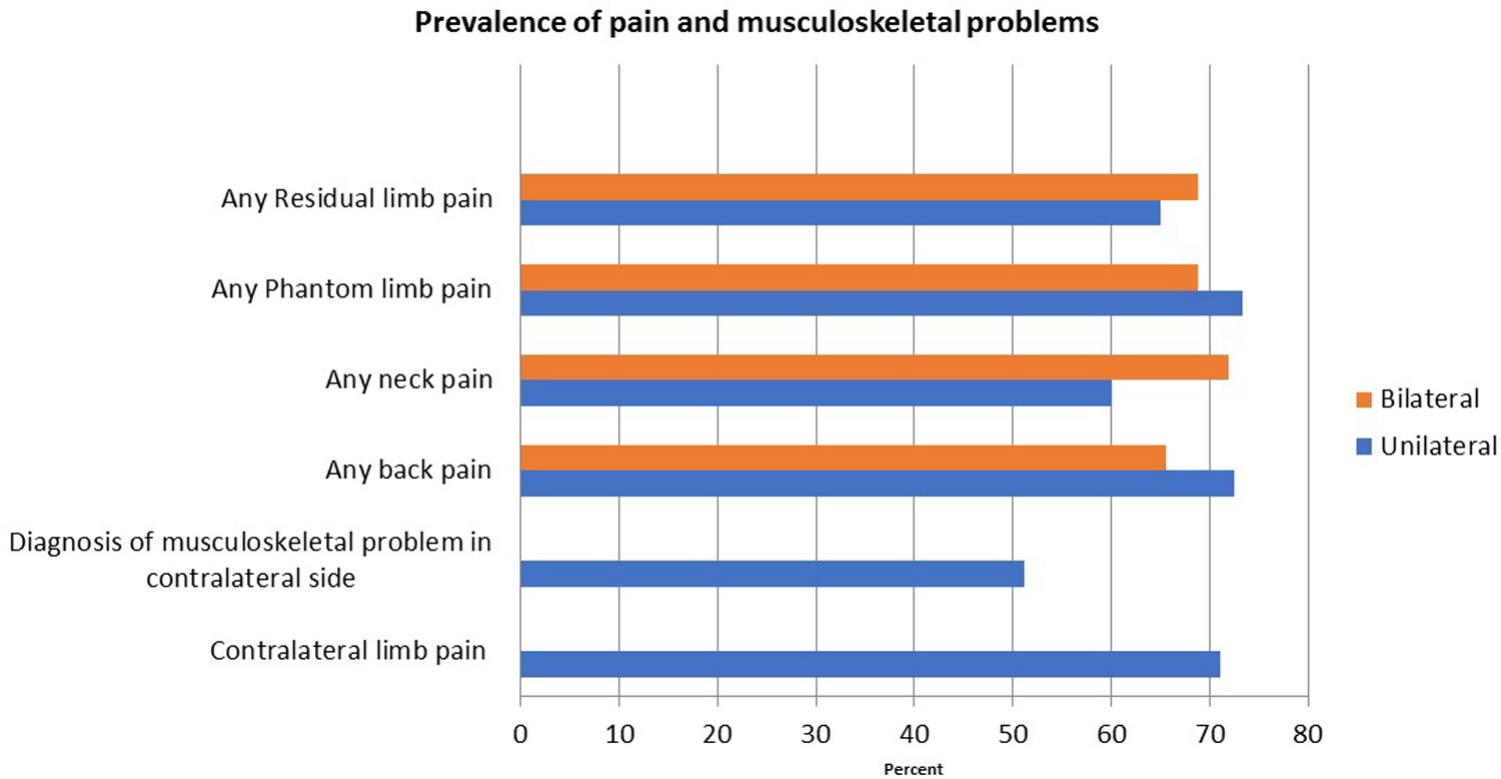
Prevalence of pain and musculoskeletal problems.

Table 6 shows the comparisons of disability and quality of life ratings for unilateral and bilateral amputees. Bilateral amputees were more disabled as measured by the QuickDASH as compared to unilateral amputees ((mean 49.5(20.7) vs. 34.7(22.0), P = 0.053)); while the t-test was not statistically significant, the Wilcoxon rank sum test indicated strong statistical significance. Scores of the VR 12 PCS and MCS did not differ by group, but PCS were lower than population norms. There were no other statistically significant differences between unilateral and bilateral amputees. Seventy one percent of unilateral amputees reported contralateral limb pain (Fig 6), and 51.2% reported at least one of the musculoskeletal conditions we asked about. In terms of pain, 72.5% of unilateral and 65.6% of bilateral amputees reported any back pain, while 60.1% of unilateral and 71.9% of bilateral amputees reported any neck pain. Phantom and residual limb pain were prevalent with 73.4% of unilateral and 68.8% of bilateral amputees reporting phantom pain and 65.0% of unilateral and 68.8% of bilateral amputees reporting any residual limb pain.

**Table 6:** Disability and quality of life.

Full sample		Unilateral		Bilateral	T-test	WRS+
	N	Mean (sd)	N	Mean (sd)	p-value	p-value
**QuickDASH**	743	34.7 (22.0)	32	49.5 (20.7)	0.0529	**0.0048**
**VR-12 PCS**	727	45.1 (8.7)	31	44.6 (9.7)	0.7648	0.7128
**VR-12 MCS**	727	49.6 (13.4)	31	50.6 (13.4)	0.6857	0.5727
**Pain and musculoskeletal conditions**	**N**	**N (%)**	**N**	**N (%)**	**Chisq p**	**Fisher’s Exact p**
Any contralateral limb pain (unilateral only)	757	538 (71.1)				
Any problem of sound side (U_B9)	771	395 (51.2)				
Any back pain	760	551 (72.5)	32	21 (65.6)	0.3950	0.4215
Any neck pain	760	457 (60.1)	32	23 (71.9)	0.1829	0.2011
Any Phantom limb pain	756	555 (73.4)	32	22 (68.8)	0.5596	0.5453
**Those with amputations distal to shoulder**		Unilateral		Bilateral		
Any Residual limb pain	663	431 (65.0)	32	22 (68.8)	0.6643	0.7091

+WRS **=** Wilcoxon rank sum test

### 3.6—Amputation care

The majority of respondents had been to a VA medical center for their amputation related care (81.8% unilateral, 84.4% bilateral) (Table 7). Fifty-seven percent of unilateral amputees and 81.3% of bilateral amputees had been to a VA amputation clinic at some time. Sixty-six percent of unilateral amputees who were prosthesis users and 80% of bilateral amputees who were prosthesis users had been to a VA amputee clinic between 2015 and the time of survey.

**Table 7:** Amputation care.

	**Unilateral** **N = 776**	**Bilateral** **N = 32**
	**N (%)**	**N (%)**
**Location of amputation-related care (ever) (all that apply)**		
VA medical center	617 (81.8)	27 (84.4)
Local prosthetist office	462 (61.3)	23 (71.9)
Non-VA health care center or hospital	280 (37.1)	17 (53.1)
Department of Defense medical center	147 (19.5)	10 (31.3)
Someplace else	94 (12.5)	5 (15.6)
Missing (n)	22	0
**Ever been to VA Amputation Clinic?**		
Yes	428 (56.8)	26 (81.3)
No	280 (37.1)	5 (15.6)
Unknown	46 (6.1)	1 (3.1)
Missing (n)	22	0
**Ever been to DoD Amputation Clinic?**		
Yes	109 (14.5)	7 (21.9)
No	601 (79.8)	21 (65.6)
Unknown	43 (5.7)	4 (12.5)
Missing (n)	22	0
**Among those who have been to VA Amputation Clinic (n = 384)**	**Unilateral** **N = 428**	**Bilateral** **N = 26**
**Year of last visit to VA Amputation clinic**		
**2008 or before**	63 (16.4)	1 (4.0)
2009	2 (0.5)	0 (0.0.)
2010	10 (2.6)	0 (0.0.)
2011	5 (1.3)	0 (0.0.)
2012	14 (3.7)	1 (4.0)
2013	12 (3.1)	1 (4.0)
2014	24 (6.3)	1 (4.0)
2015	40 (10.4)	7 (28.0)
2016	78 (20.3)	2 (8.0)
2017	127 (33.1)	1 (44.0)
2018	9 (2.3)	0 (0.0.)
Missing (n)	44	1

## 4.0—Discussion

We conducted the first-of-its-kind national study of Veterans with major upper limb loss. Our study was by far the largest study of Veterans with upper limb amputation conducted to date, and its sampling strategy and analytical methods make the results generalizable to Veterans with upper limb amputation who were seen at the VA for care. We characterized amputation level and etiology, prosthesis and terminal device types used, prosthesis suspension methods, as well amount and frequency of prosthesis use and prosthesis training receipt. For persons who had abandoned using a prosthesis, we also described the reasons for abandonment by device type. Additionally, we compared prosthetic satisfaction, and several measures of health-related quality of life of unilateral and bilateral amputees.

Sixty percent of unilateral amputees in our study were prosthesis users, fewer than that reported for combat Veterans in earlier studies (72% Vietnam and 76% OEF/OIF).[17] We found that 84% of our respondents with bilateral amputation were prosthesis users, a similar prevalence to that reported in OEF/OIF combat amputees with bilateral upper limb amputation (85.7%).[17] We also found that 6.8% of unilateral amputees had never received a prosthesis, a slighter higher prevalence than reported in combat amputees.[17] Differences in prevalence of prosthesis use in our sample compared to earlier reports may be related to amputation level of respondents as well as etiology of amputation. Twelve percent of our sample were amputees with shoulder or forequarter amputation (107 persons) (compared to 4.4% in the earlier study), and included 55 persons with elbow disarticulation, and 132 persons with wrist disarticulation (higher proportions in our sample than in the earlier study). Elbow disarticulation and wrist disarticulation may be particularly challenging to fit with prostheses. Our sample included amputees with any type of etiology (only 35% were combat amputees). These factors may explain differences in prosthesis use. Future analyses of our data will explore these and additional factors that may be associated with prosthesis use and abandonment.

The majority of prosthesis users in our study used body powered devices (70.9% of unilateral amputees, and 77.8% of bilateral amputees) as their primary device. This finding is consistent with earlier reports that only 8% of unilateral combat amputees from Vietnam had ever received a myoelectric device. We found that 42% of unilateral amputees used more than one type of terminal device. Only 10.9% of unilateral amputees and no bilateral amputees used a multi-degree of freedom powered terminal device as their primary terminal devices. We plan to explore the relationship between type of devices used and functional abilities in future analyses.

A majority of respondents had abandoned a prosthesis at some point, and the most common reasons for abandonment were lack of function, problems with fit/comfort and too much fuss. There were some differences between reasons for abandonment of devices by unilateral and bilateral amputees. No bilateral amputees reported abandoning a body powered device because it was too heavy or fatiguing.

Almost 30% of unilateral amputees and 41% of bilateral amputees who had ever used a prosthesis had not received any training to use their first prosthesis, and 34% of unilateral and 48.0% (on left) and 44.4% (on right) of bilateral amputees had not received training to use their current prosthesis. The amount of prosthetic training received varied considerably, with a greater proportion of bilateral amputees having had 30 or more training visits. To our knowledge, ours is the first study that has examined receipt of prosthetic training. Prosthetic training is considered critical for maximizing functional capabilities with the prosthesis. [28–30] The impact of prosthetic training receipt on function and disability, and prosthesis abandonment has not been fully examined, and is another area that we plan to explore using our survey data.

We quantified the hours of prosthesis use per day and found that 28.2% of unilateral amputees used their devices four hours or less per day, and only 52.1% used them 8 hours or more. In contrast, 76% bilateral amputees used at least one of their prosthesis 8 hours per day or more. Historically, some have defined full time prosthesis use as use of at least 8 hours per day.[4] Our findings on prosthesis use point to the need for future studies to examine the relationship between prosthesis satisfaction and hours of use, and between self-rated disability and hours of prosthesis use. These analyses will be possible in future studies using our data.

Generally, Veterans responses indicated that they were neutral or somewhat satisfied with their prostheses as measured by the TAPES satisfaction measure and the OPUS CSD scores. Items with the lowest satisfaction ratings included comfort (TAPES), and self-consciousness about the prosthesis, wear and tear of clothing and device costs (OPUS). The unilateral values for the CSD in our sample fall between the 64-71^st^ percentile, while the bilateral values fall between the 71-78^th^ percentile of reported provisional normative scores [19], which indicates lower than average satisfaction with devices in our sample. It is difficult to make other direction comparisons between our findings on prosthesis satisfaction and those reported in prior studies of combat amputees that employed modified scales and used dichotomous scoring. [17, 31] The OIG reported that 69.6% of traumatic upper limb amputees in their study were satisfied were their prostheses.

The QuickDASH measure of self-reported disability showed that Veterans with upper limb amputation have significant disability as compared to normative values. [32] Not surprisingly, bilateral amputees rated themselves as more disabled as compared to unilateral amputees (49.5 vs 34.7), (WSR P<0.01) Scores for unilateral amputees were comparable to those reported in a prior OIG report of OEF/OIF combat Veterans with upper limb amputation, (mean 36.6, 95 percent CI: 31.6, 41.6).[31] Future analyses from our data will examine the impact of QuickDASH scores, amputation and prosthesis characteristics on the need for and amount of help with daily activities.

The VR-12 PCS scores in our sample were approximately 0.5 standard deviation below population means (for non-disabled), indicating moderately impaired physical functioning, with no large differences between unilateral and bilateral amputee groups. In contrast, the VR-12 MCS scores were at the population mean, indicating normal mental/emotional functioning. These findings are consistent with prior reports of lower physical functioning, and greater pain [33] and equivalent mental health for upper limb amputees. [34]

We found that the majority (71%) of unilateral amputees reported that they had at least one type of musculoskeletal condition of the contralateral limb, and that the majority of respondents reported back and neck pain. These prevalence rates are higher than reported in Norwegian upper limb amputees. [35, 36] In the Norwegian sample, the prevalence rate of musculoskeletal conditions in persons with upper limb amputation was about twice that of the general population; we do not have comparable data to know how prevalence of back and neck pain in our sample compare to an age-matched Veteran population. In addition, the relationship between contralateral limb pain, back and neck pain and years of prosthesis use as well as type of prosthesis used is not known. These relationships can be explored in future research using our data.

Phantom and residual limb pain were also prevalent in Veterans with upper limb amputation. Phantom limb pain impacted almost three quarters of unilateral amputees and 69% of bilateral amputees, while residual limb pain was reported by approximately two thirds of respondents. These rates are higher than reported in prior literature (42.6% phantom pain, 43% residual limb pain).[37] Future studies will examine the factors associated with prevalent phantom and residual limb pain.

Given that the sampling frame was drawn from Veterans who had received some type of care at the VA, it is not surprising that the majority of respondents had been to the VA for amputation related care, with a higher proportion of bilateral amputees (81.3%) as compared to unilateral amputees (57%) having gone to a VA amputation clinic. This finding makes some sense, given the greater complexity in meeting the needs of bilateral amputees. While the vast majority of prosthesis users had their last visit to a VA amputation clinic since 2015, about 26.3% of unilateral and 16% of bilateral had not been to an amputation clinic in the previous 5 years. Further study is needed to understand the impact of site of amputation care on prosthetic satisfaction and other important outcomes.

### 4.1—Limitations

We observed minor differences in survey respondents and non-respondents. Respondents were an average of 1.8 years younger than non-respondents, females were more likely to respond then males, and a slightly higher proportion of respondents had been seen at the VA in the years 2015 and 2016. We believe that these differences are small, and given the strong overall response rate, our findings are generalizable to Veterans with upper limb amputation who received care in the VA between 2011-2015.The results may have limited generalizability to Veterans who received care only after 2015 (and thus were not identified in our original sampling frame), and to the overall civilian population with limb loss. Our sampling frame was generated from VA medical record data, using inclusive criteria for identifying upper limb amputees (any instance of a diagnosis). We found that 829 persons, about 15% of the sample were not upper limb amputees, as identified through opt outs and after screening. We can assume that a similar proportion of persons with unknown eligibility were also not amputees (157 persons). Thus, the total estimate of persons without amputation would be 986, or approximately 17% of the original sampling frame. Our study response rate was calculated using the American Association of Public Opinion Research methodology. [27] Using this methodology, we estimated a proportion of non-respondents as being ineligible; if we considered them all to be eligible, the response rate would be 33%.

We do not believe that misclassification errors from medical records are unique to the diagnosis of upper limb amputation, but we do not have any comparative data. Given the possibility of medical coding errors, it is possible that there were additional Veterans with major upper limb amputation who were not coded as such and thus did not appear in our sampling frame. However, we have no visibility into the prevalence of missing amputation diagnosis codes.

Our survey instrument was long, and it is possible that some respondents became fatigued during interviews, however we have no way of knowing to what extent this may have influenced accuracy of data collection. We had very few interviews that were cut short. Although we compared outcomes of unilateral and bilateral amputees statistically, our sample of bilateral amputees was small (N = 32). This sample size limited us in detecting minor differences as statistically significant when they may have existed. That said, we were adequately powered to detect moderate differences between unilateral and bilateral amputee groups. We conducted a post-hoc power analysis for each outcome measure that we compared, utilizing the standard deviations of the measure and the sample size for each group. We had at least 80% power to detect moderate differences in group means of approximately 0.5 sd (effect sizes 0.51–0.65) for the QuickDASH, VR12 MCS and PCS, TAPES, and OPUS CSD. It is possible that smaller differences between groups actually existed, but that we are underpowered to detect them.

Although we attempted to compare OPUS CSD findings to normative values reported in 2010 to assist in interpreting the scores, these comparisons must be interpreted cautiously. Normative values for the OPUS were drawn from work with predominantly lower limb amputees and most of the data were from international samples. Therefore, we are unsure how our OPUS CSD results would compare to those from upper limb amputees in the U.S and/or who were non-Veterans. Further, we are unable to compare our prosthetic satisfaction results to those reported for Vietnam and OEF/OIF combat amputees because prior analyses used modified satisfaction items, created a new satisfaction scale, and dichotomized responses of individual items.

## 5.0—Conclusions

This paper reports summary findings from the first ever nationally representative study of Veterans with all cause upper limb amputation, and one of the largest studies to describe upper limb amputees, their prosthesis use, satisfaction with devices, health-related quality of life and care receipt. We found that rates of prosthesis use were lower than reported in samples of combat Veterans.[13] Body powered devices were used by 70.9% of unilateral and 76.0% (on left) and 77.8% (on right) of bilateral amputees. Multi-degree of freedom terminal devices, used by 11% of unilateral amputees, were not used by any bilateral amputees. Overall, we found that Veterans who were prosthesis users were somewhat satisfied with their devices, although only 52% utilized their devices at least 8 hours per day and substantial proportion used them less than 2 hours per day. A substantial proportion of respondents had not received any training to use either their initial prostheses, or their current prostheses.

Veterans with upper limb amputation rated themselves as disabled on the QuickDASH, and were found to have moderately impaired physical functioning as measured by the VR-12. Musculoskeletal problems, phantom limb and residual limb pain affected the majority, with rates of phantom and residual limb pain higher than previously reported. [37] A substantial proportion of Veterans did not receive amputation related care at the VA amputation care and many had never been to a VA amputation clinic, suggesting an opportunity to increase access to care.

## Supporting information

S1 Appendix Amputation codes. (DOCX)

S2 Appendix Copy of survey instrument. (DOCX)
