# Supplementary material for: A national study of Veterans with major upper limb amputation: Survey methods, participants, and summary findings
Source: PLoS One. 2019 Mar 14;14(3):e0213578. doi: 10.1371/journal.pone.0213578 (PMC6417699; doi:10.1371/journal.pone.0213578)
Supplement: S1 Appendix — (DOCX) [file pone.0213578.s001.docx]

S1. Amputation Codes

- ICD9 Diagnosis Codes:
  - 887.0, 887.1, 887.2, 887.3, V49.64, V49.65, V49.66, V49.67, 887.4, 887.5, 887.6, 887.7, V49.6
- ICD9 Procedure Codes:
  - 84.04, 84.05, 84.06, 84.07, 84.08, 84.09, 84.00
- ICD10 Diagnosis Codes:
  - S68.411A, S68.411D, S68.411S, S68.412A, S68.412D, S68.412S, S68.419A, S68.419D, S68.419S, S68.421A, S68.421D, S68.421S, S68.422A, S68.422D, S68.422S, S68.429A, S68.429D, S68.429S, S48.011A, S48.011D, S48.011S, S48.012A, S48.012D, S48.012S, S48.019A, S48.019D, S48.019S, S48.021A, S48.021D, S48.021S, S48.022A, S48.022D, S48.022S, S48.029A, S48.029D, S48.029S, S48.111A, S48.111D, S48.111S, S48.112A, S48.112D, S48.112S, S48.119A, S48.119D, S48.119S, S48.121A, S48.121D, S48.121S, S48.122A, S48.122D, S48.122S, S48.129A, S48.129D, S48.129S, S48.911A, S48.911D, S48.911S, S48.912A, S48.912D, S48.912S, S48.919A, S48.919D, S48.919S, S48.921A, S48.921D, S48.921S, S48.922A, S48.922D, S48.922S, S48.929A, S48.929D, S48.929S, S58.011A, S58.011D, S58.011S, S58.012A, S58.012D, S58.012S, S58.019A, S58.019D, S58.019S, S58.021A, S58.021D, S58.021S, S58.022A, S58.022D, S58.022S, S58.029A, S58.029D, S58.029S, S58.111A, S58.111D, S58.111S, S58.112A, S58.112D, S58.112S, S58.119A, S58.119D, S58.119S, S58.121A, S58.121D, S58.121S, S58.122A, S58.122D, S58.122S, S58.129A, S58.129D, S58.129S, S58.911A, S58.911D, S58.911S, S58.912A, S58.912D, S58.912S, S58.919A, S58.919D, S58.919S, S58.921A, S58.921D, S58.921S, S58.922A, S58.922D, S58.922S, S58.929A, S58.929D, S58.929S, Z89.121, Z89.122, Z89.129, Z89.209, Z89.211, Z89.212, Z89.219, Z89.221, Z89.222, Z89.229, Z89.231, Z89.232, Z89.239, Z89.201, Z89.202
- ICD10 Procedure Codes
  - 0X600ZZ, 0X610ZZ, 0X620ZZ, 0X630ZZ, 0X680Z1, 0X680Z2, 0X680Z3, 0X690Z1, 0X690Z2, 0X690Z3, 0X6B0ZZ, 0X6C0ZZ, 0X6D0Z1, 0X6D0Z2, 0X6D0Z3, 0X6F0Z1, 0X6F0Z2, 0X6F0Z3, 0X6J0Z0
- CPT Procedure Codes:
  - 23900, 23920, 24900, 24920, 24925, 24930, 24931, 25900, 25905, 25907, 25909, 25915, 25920, 25924, 24935, 24940
- VETSNET Codes:
  - 5120 – Loss of arm
  - 5121 – Arm amputation above insertion of deltoid
  - 5122 – Arm amputation below insertion of deltoid
  - 5123 – Arm amputation above insertion of pronator teres
  - 5124 – Arm amputation below insertion of pronator teres

| Type | Upper Limb Amputation Codes |
| --- | --- |
| ICD9 Diagnosis Codes | 887.0, 887.1, 887.2, 887.3, V49.64, V49.65, V49.66, V49.67, 887.4, 887.5, 887.6, 887.7, V49.6 |
| ICD9 Procedure Codes | 84.04, 84.05, 84.06, 84.07, 84.08, 84.09, 84.00 |
| ICD10 Diagnosis Codes | S68.411A, S68.411D, S68.411S, S68.412A, S68.412D, S68.412S, S68.419A, S68.419D, S68.419S, S68.421A, S68.421D, S68.421S, S68.422A, S68.422D, S68.422S, S68.429A, S68.429D, S68.429S, S48.011A, S48.011D, S48.011S, S48.012A, S48.012D, S48.012S, S48.019A, S48.019D, S48.019S, S48.021A, S48.021D, S48.021S, S48.022A, S48.022D, S48.022S, S48.029A, S48.029D, S48.029S, S48.111A, S48.111D, S48.111S, S48.112A, S48.112D, S48.112S, S48.119A, S48.119D, S48.119S, S48.121A, S48.121D, S48.121S, S48.122A, S48.122D, S48.122S, S48.129A, S48.129D, S48.129S, S48.911A, S48.911D, S48.911S, S48.912A, S48.912D, S48.912S, S48.919A, S48.919D, S48.919S, S48.921A, S48.921D, S48.921S, S48.922A, S48.922D, S48.922S, S48.929A, S48.929D, S48.929S, S58.011A, S58.011D, S58.011S, S58.012A, S58.012D, S58.012S, S58.019A, S58.019D, S58.019S, S58.021A, S58.021D, S58.021S, S58.022A, S58.022D, S58.022S, S58.029A, S58.029D, S58.029S, S58.111A, S58.111D, S58.111S, S58.112A, S58.112D, S58.112S, S58.119A, S58.119D, S58.119S, S58.121A, S58.121D, S58.121S, S58.122A, S58.122D, S58.122S, S58.129A, S58.129D, S58.129S, S58.911A, S58.911D, S58.911S, S58.912A, S58.912D, S58.912S, S58.919A, S58.919D, S58.919S, S58.921A, S58.921D, S58.921S, S58.922A, S58.922D, S58.922S, S58.929A, S58.929D, S58.929S, Z89.121, Z89.122, Z89.129, Z89.209, Z89.211, Z89.212, Z89.219, Z89.221, Z89.222, Z89.229, Z89.231, Z89.232, Z89.239, Z89.201, Z89.202 |
| ICD10 Procedure Codes | 0X600ZZ, 0X610ZZ, 0X620ZZ, 0X630ZZ, 0X680Z1, 0X680Z2, 0X680Z3, 0X690Z1, 0X690Z2, 0X690Z3, 0X6B0ZZ, 0X6C0ZZ, 0X6D0Z1, 0X6D0Z2, 0X6D0Z3, 0X6F0Z1, 0X6F0Z2, 0X6F0Z3, 0X6J0Z0 |
| CPT Procedure Codes | 23900, 23920, 24900, 24920, 24925, 24930, 24931, 25900, 25905, 25907, 25909, 25915, 25920, 25924, 24935, 24940 |
| VETSNET Codes | 5120 – Loss of arm  5121 – Arm amputation above insertion of deltoid  5122 – Arm amputation below insertion of deltoid  5123 – Arm amputation above insertion of pronator teres  5124 – Arm amputation below insertion of pronator teres |
