# Supplementary material for: A national study of Veterans with major upper limb amputation: Survey methods, participants, and summary findings
Source: PLoS One. 2019 Mar 14;14(3):e0213578. doi: 10.1371/journal.pone.0213578 (PMC6417699; doi:10.1371/journal.pone.0213578)
Supplement: S2 Appendix — (DOCX) [file pone.0213578.s002.docx]

**Needs, Preferences and Functional Abilities of Veterans and Service Members with Upper Limb Amputation:** Unilateral Amputees Survey

**Part A: Screener**

**[ASK ALL]**

A1. Do you have an upper limb amputation at the wrist or higher?

**[INTERVIEWER NOTE:** IF DK/NOT SURE/REFUSED, PROMPT: It is very important for you to answer this question since it will help determine if you’re eligible for the study.**]**

1 Yes

2 No

98 DON’T KNOW/NOT SURE [DO NOT READ]

99 REFUSED [DO NOT READ]

**[ASK IF A1=1 (YES, HAS UPPER LIMB AMPUTATION)]**

A2. On which side is your upper limb amputation? Please select one response.

**[INTERVIEWER NOTE:** IF DK/NOT SURE/REFUSED, PROMPT: It is very important for you to answer this question since it will help determine if you’re eligible for the study.**]**

1 Right side

2 Left side

3 Both sides

98 DON’T KNOW/NOT SURE [DO NOT READ]

99 REFUSED [DO NOT READ]

**[ASK IF A1=1 (YES, HAS UPPER LIMB AMPUTATION)]**

A3. Do you have an amputation of one or more lower limbs?

1 Yes

2 No

98 DON’T KNOW/NOT SURE [DO NOT READ]

99 REFUSED [DO NOT READ]

**[ASK IF A3=1 (YES, HAS LOWER LIMB AMPUTATION)]**

A4. On which side is your lower limb amputation? Please select one response.

1 Right side

2 Left side

3 Both sides

98 DON’T KNOW/NOT SURE [DO NOT READ]

99 REFUSED [DO NOT READ]

**[ASK IF A1=1 (YES, HAS UPPER LIMB AMPUTATION)]**

A5. Are you a Veteran or Active Service Member?

1 Yes

2 No

98 DON’T KNOW/NOT SURE [DO NOT READ]

99 REFUSED [DO NOT READ]

**Part B: Amputation History**

U_B_INTRO. The first questions are about your upper limb amputation.

**[ASK ALL]**

U_B1. At what level is your upper limb amputation?

1 Chest wall level, sometimes called “forequarter”

2 At the shoulder joint

3 Above the elbow

4 At the elbow

5 Below the elbow

6 At the wrist joint

7 Through the hand

98 DON’T KNOW/NOT SURE [DO NOT READ]

99 REFUSED [DO NOT READ]

**[ASK ALL]**

U_B2a. What was the month and year of your initial amputation?

MONTH:______

98 DON’T KNOW/NOT SURE [DO NOT READ]

99 REFUSED [DO NOT READ]

**[ASK ALL]**

U_B2b.

**[INTERVIEWER NOTE:** ENTER 98 FOR DON’T KNOW AND 99 FOR REFUSED**]**

YEAR: ______

**[ASK ALL] [GRID; MP DOWN, SP ACROSS]**

U_B3. Which of the following were the cause of your initial amputation?

**[INTERVIEWER NOTE:** REPEAT RESPONSE OPTIONS AS NECESSARY**]**

|  | Yes | No | DON’T KNOW/NOT SURE [DO NOT READ] | REFUSED [DO NOT READ] |
| --- | --- | --- | --- | --- |
| a. Combat injury? [Yes or No?] | 1 | 2 | 98 | 99 |
| b. Accident? | 1 | 2 | 98 | 99 |
| c. Burn? | 1 | 2 | 98 | 99 |
| d. Cancer? | 1 | 2 | 98 | 99 |
| e. Diabetes? | 1 | 2 | 98 | 99 |
| f. Infection? | 1 | 2 | 98 | 99 |

| g. Some other reason? [IF YES: Please explain] | 1 | 2 | 98 | 99 |
| --- | --- | --- | --- | --- |

**[IF U_B3g=1 (SOME OTHER REASON)] [TEXT BOX]**

U_B3_SPECIFY. ________________ __________________

**[ASK IF U_B3a OR U_B3b=1 (YES)]**

U_B4. How long was the time between your initial injury and the amputation surgery?

1 Did the amputation occur on the same day as the injury?

2 Within 1 month of the injury?

3 1 to less than 3 months?

4 3 to less than 6 months?

5 6 to less than 12 months?

6 12 months or more?

98 DON’T KNOW/NOT SURE [DO NOT READ]

99 REFUSED [DO NOT READ]

**[ASK ALL]**

U_B5. What was your dominant hand before the amputation?

**[INTERVIEWER NOTE:** DO NOT READ UNLESS NECESSARY**]**

1 LEFT

2 RIGHT

3 BOTH

98 DON’T KNOW/NOT SURE [DO NOT READ]

99 REFUSED [DO NOT READ]

**[ASK ALL]**

U_B6. What is your dominant hand now?

**[INTERVIEWER NOTE:** DO NOT READ UNLESS NECESSARY**]**

1 LEFT

2 RIGHT

3 BOTH

98 DON’T KNOW/NOT SURE [DO NOT READ]

99 REFUSED [DO NOT READ]

**[ASK ALL]**

U_B7. Have you had targeted muscle reinnervation (TMR) surgery, which is a surgical procedure that moves nerves to new locations to make it easier to control a prosthesis?

**[INTERVIEWER NOTE:** DO NOT READ UNLESS NECESSARY**]**

1 YES

2 NO

98 DON’T KNOW/NOT SURE [DO NOT READ]

99 REFUSED [DO NOT READ]

**[ASK ALL]**

U_B8. Thinking of your arm that is not amputated, do you experience any of the following in your hand, wrist or elbow?

**[INTERVIEWER NOTE:** REPEAT RESPONSE OPTIONS AS NECESSARY**]**

|  | Yes | No | DON’T KNOW/NOT SURE [DO NOT READ] | REFUSED [DO NOT READ] |
| --- | --- | --- | --- | --- |
| a. Loss of sensation? [Yes or No?] | 1 | 2 | 98 | 99 |
| b. Loss of movement? | 1 | 2 | 98 | 99 |
| c. Weakness? | 1 | 2 | 98 | 99 |
| d. Pain? | 1 | 2 | 98 | 99 |

**[ASK ALL]**

U_B9. Have you ever been diagnosed with a problem in the arm that is not amputated?

**[INTERVIEWER NOTE:** DO NOT READ UNLESS NECESSARY**]**

1 YES

2 NO

98 DON’T KNOW/NOT SURE [DO NOT READ]

99 REFUSED [DO NOT READ]

**[ASK IF U_B9=1 (YES)]**

U_B10. Which of the following problems have you been diagnosed with? Please answer Yes or No for each problem I read.

**[INTERVIEWER NOTE**: Read information in parentheses only if respondent requests clarification**]**

**[INTERVIEWER NOTE:** REPEAT RESPONSE OPTIONS AS NECESSARY**]**

|  | Yes | No | DON’T KNOW/NOT SURE [DO NOT READ] | REFUSED [DO NOT READ] |
| --- | --- | --- | --- | --- |
| a. Carpal tunnel syndrome in the wrist [Yes or No?] | 1 | 2 | 98 | 99 |
| b. Tendonitis in the wrist | 1 | 2 | 98 | 99 |
| c. Tendonitis of the elbow (lateral or medial epicondylitis) | 1 | 2 | 98 | 99 |
| d. Tendonitis of the finger (Stenosing Tenosynovitis) | 1 | 2 | 98 | 99 |
| e. Tendonitis of the thumb | 1 | 2 | 98 | 99 |
| f. Ganglion cyst | 1 | 2 | 98 | 99 |
| g. Rotator cuff tendonitis in the shoulder | 1 | 2 | 98 | 99 |
| h. Osteoarthritis / degenerative joint disease | 1 | 2 | 98 | 99 |
| i. Nerve damage | 1 | 2 | 98 | 99 |

| j. Some other problem [IF YES: Please explain] | 1 | 2 | 98 | 99 |
| --- | --- | --- | --- | --- |

**[IF U_B10j=1 (SOME OTHER PROBLEM)] [TEXT BOX]**

U_B10_SPECIFY. __________________________________________

**[ASK ALL]**

U_B11. Do you need help from another person to do daily activities such as feeding, dressing, transfers and walking?

**[INTERVIEWER NOTE:** DO NOT READ UNLESS NECESSARY**]**

1 YES

2 NO

98 DON’T KNOW/NOT SURE [DO NOT READ]

99 REFUSED [DO NOT READ]

**[ASK IF U_B11=1 (YES)]**

U_B12. How many hours in a typical day do you need help?

**[INTERVIEWER NOTE:** DO NOT READ UNLESS NECESSARY**]**

1 Less than 1 hour

2 1 to 2 hours

3 3 to 4 hours

4 5 to 6 hours

5 7 to 8 hours

6 More than 8 hours

98 DON’T KNOW/NOT SURE [DO NOT READ]

99 REFUSED [DO NOT READ]

**Part C: Prosthetic Use**

U_C_INTRO. Next, we are interested in learning about the types and number of prostheses you have ever used.

**[ASK ALL]**

U_C1. Have you ever used a prosthesis?

**[INTERVIEWER NOTE:** DO NOT READ UNLESS NECESSARY**]**

1 YES

2 NO

98 DON’T KNOW/NOT SURE [DO NOT READ]

99 REFUSED [DO NOT READ]

**[ASK IF U_C1=2 (NO, NEVER USED PROSTHESIS)]**

U_C1a. Please explain why you have never used a prosthesis.

**­­____________________________________________________________________**

**[ASK IF U_C1=1 (YES, HAS USED PROSTHESIS)]**

U_C2. About how long after your amputation did you receive your first prosthesis?

1 In less than 3 months

2 3 months to less than 6 months

3 6 months to less than 12 months

4 12 months or more

98 DON’T KNOW/NOT SURE [DO NOT READ]

99 REFUSED [DO NOT READ]

**[ASK IF U_C1=1 (YES, HAS USED PROSTHESIS)]**

U_C3. What type of device was your first prosthesis?

**[INTERVIEWER NOTE:** if respondent says “Battery powered” which is not on the list, code as myoelectric.**]**

1 Body powered

2 Myoelectric

3 Hybrid

4 Cosmetic

5 Sport (recreation)

98 DON’T KNOW/NOT SURE [DO NOT READ]

99 REFUSED [DO NOT READ]

**[ASK IF U_C1=1 (YES, HAS USED PROSTHESIS)]**

U_C4. Did you receive training to use your prosthesis immediately after you received your first prosthesis?

**[INTERVIEWER NOTE:** DO NOT READ UNLESS NECESSARY**]**

1 YES

2 NO

98 DON’T KNOW/NOT SURE [DO NOT READ]

99 REFUSED [DO NOT READ]

**[ASK IF U_C1=1 (YES, HAS USED PROSTHESIS)]**

U_C5. Do you currently use a prosthesis?

**[INTERVIEWER NOTE:** DO NOT READ UNLESS NECESSARY**]**

1 YES

2 NO

98 DON’T KNOW/NOT SURE [DO NOT READ]

99 REFUSED [DO NOT READ]

**[ASK IF U_C5=1 (YES, CURRENTLY USES PROSTHESIS)]**

U_C6. How many hours a day do you typically use a prosthesis?

1 Less than 2 hours a day

2 2 to less than 4 hours a day

3 4 to less than 8 hours a day

4 8 to less than 12 hours a day

5 12 hours or more a day

98 DON’T KNOW/NOT SURE [DO NOT READ]

99 REFUSED [DO NOT READ]

**[ASK IF U_C5=1 (YES, CURRENTLY USES PROSTHESIS)]**

U_C7. When did you get your most recent prosthesis?

1 Less than 3 months ago

2 3 months to less than 6 months ago

3 6 months to less than 1 year ago

4 1 year to less than 2 years ago

5 2 years to less than 4 years ago

6 4 years ago or longer

98 DON’T KNOW/NOT SURE [DO NOT READ]

99 REFUSED [DO NOT READ]

**[ASK IF U_C5=1 (YES, CURRENTLY USES PROSTHESIS)]**

U_C8. How many different types of prostheses do you currently use?

**[INTERVIEWER NOTE:** DO NOT READ UNLESS NECESSARY**]**

1 ONE

2 TWO OR MORE

98 DON’T KNOW/NOT SURE [DO NOT READ]

99 REFUSED [DO NOT READ]

**[ASK IF U_C8=1 (ONE TYPE OF PROSTHESIS)]**

U_C9. What type of prosthesis do you currently use?

**[INTERVIEWER NOTE:** if respondent says “Battery powered” which is not on the list, code as myoelectric.**]**

1 Body powered

2 Myoelectric

3 Hybrid

4 Cosmetic

5 Sport

98 DON’T KNOW/NOT SURE [DO NOT READ]

99 REFUSED [DO NOT READ]

**[ASK IF U_C8=1 (ONE TYPE OF PROSTHESIS)]**

U_C10. How is your prosthesis attached to your body? Please answer Yes or No for each item I read.

**[INTERVIEWER NOTE:** IF PARTICIPANT IS CONFUSED BY THIS QUESTION, IT SHOULD BE EXPLAINED THAT THE QUESTION IS ABOUT HOW THE PROSTHESIS SOCKET IS ATTACHED.**]**

**[INTERVIEWER NOTE:** REPEAT RESPONSE OPTIONS AS NECESSARY**]**

|  | Yes | No | DON’T KNOW/NOT SURE [DO NOT READ] | REFUSED [DO NOT READ] |
| --- | --- | --- | --- | --- |
| a. Suction [Yes or No?] | 1 | 2 | 98 | 99 |
| b. Gel or silicone liner with pin | 1 | 2 | 98 | 99 |
| c. Vacuum | 1 | 2 | 98 | 99 |
| d. Self-suspending because of the socket shape | 1 | 2 | 98 | 99 |
| e. Harnessing | 1 | 2 | 98 | 99 |
| f. External suspension sleeve | 1 | 2 | 98 | 99 |

**[ASK IF U_C8=1 (ONE TYPE OF PROSTHESIS)]**

U_C11. How often do you use this device? Would you say…

1 Daily

2 Weekly

3 Monthly

4 Every few months

5 1 to 2 times per year

98 DON’T KNOW/NOT SURE [DO NOT READ]

99 REFUSED [DO NOT READ]

**[ASK IF U_C8=2 (TWO OR MORE TYPES OF PROSTHESES)]**

U_C12. Which type of prosthesis do you consider your primary device? That is the one you use the most hours and the most often.

**[INTERVIEWER NOTE:** if respondent says “Battery powered” which is not on the list, code as myoelectric.**]**

1 Body powered

2 Myoelectric

3 Hybrid

4 Cosmetic

5 Sport

98 DON’T KNOW/NOT SURE [DO NOT READ]

99 REFUSED [DO NOT READ]

**[ASK IF U_C8=2 (TWO OR MORE TYPES OF PROSTHESES)]**

U_C13. How is this prosthesis attached to your body? Please answer Yes or No for each item I read.

**[INTERVIEWER NOTE:** REPEAT RESPONSE OPTIONS AS NECESSARY**]**

|  | Yes | No | DON’T KNOW/NOT SURE [DO NOT READ] | REFUSED [DO NOT READ] |
| --- | --- | --- | --- | --- |
| a. Suction [Yes or No?] | 1 | 2 | 98 | 99 |
| b. Gel or silicone liner with pin | 1 | 2 | 98 | 99 |
| c. Vacuum | 1 | 2 | 98 | 99 |
| d. Self-suspending because of the socket shape | 1 | 2 | 98 | 99 |
| e. Harnessing | 1 | 2 | 98 | 99 |
| f. External suspension sleeve | 1 | 2 | 98 | 99 |

**[ASK IF U_C8=2 (TWO OR MORE TYPES OF PROSTHESES)]**

U_C14. How often do you use this prosthesis? Would you say…

1 Daily

2 Weekly

3 Monthly

4 Every few months

5 1 to 2 times per year

98 DON’T KNOW/NOT SURE [DO NOT READ]

99 REFUSED [DO NOT READ]

**[ASK IF U_C8=2 (TWO OR MORE TYPES OF PROSTHESES)]**

U_C15. Which type of prosthesis do you consider your secondary or spare device? That is the one you use the next most often.

**[INTERVIEWER NOTE:** if respondent says “Battery powered” which is not on the list, code as myoelectric.**]**

1 Body powered

2 Myoelectric

3 Hybrid

4 Cosmetic

5 Sport

98 DON’T KNOW/NOT SURE [DO NOT READ]

99 REFUSED [DO NOT READ]

**[ASK IF U_C8=2 (TWO OR MORE TYPES OF PROSTHESES)]**

U_C16. How often do you use this prosthesis? Would you say…

1 Daily

2 Weekly

3 Monthly

4 Every few months

5 1 to 2 times per year

98 DON’T KNOW/NOT SURE [DO NOT READ]

99 REFUSED [DO NOT READ]

**[ASK IF U_C5=1 (YES, CURRENTLY USES PROSTHESIS)]**

U_C17. How many different types of terminal devices do you currently use?

**[INTERVIEWER NOTE:** Respondents should know what a terminal device is, but just in case: It is the end of the prosthesis - the hook or the hand.**]**

**[INTERVIEWER NOTE:** DO NOT READ UNLESS NECESSARY**]**

1 ONE

2 TWO OR MORE

98 DON’T KNOW/NOT SURE [DO NOT READ]

99 REFUSED [DO NOT READ]

**[ASK IF U_C17=1 (ONE TYPE OF TERMINAL DEVICE)]**

U_C18. What type of terminal device do you currently use?

1 Hook with voluntary opening

2 Hook with voluntary closing

3 Greiffer

4 Powered Hook (ETD)

5 Sensor Speed Hand

6 I-limb

7 Michaelangelo Hand

8 Bebionic Hand

9 Some other device [Please explain]

98 DON’T KNOW/NOT SURE [DO NOT READ]

99 REFUSED [DO NOT READ]

**[ASK IF U_C18=9 (SOME OTHER DEVICE)]**

U_C18_SPECIFY. ___________________________________

**[ASK IF U_C17=2 (TWO OR MORE TYPES OF TERMINAL DEVICE)]**

U_C19. Which type of terminal device do you consider your primary device? That is the one you use most often.

1 Hook with voluntary opening

2 Hook with voluntary closing

3 Greiffer

4 Powered Hook (ETD)

5 Sensor Speed Hand

6 I-limb

7 Michaelangelo Hand

8 Bebionic Hand

9 Some other device [Please explain]

98 DON’T KNOW/NOT SURE [DO NOT READ]

99 REFUSED [DO NOT READ]

**[ASK IF U_C19=9 (SOME OTHER DEVICE)]**

U_C19_SPECIFY. ___________________________________

**[ASK IF U_C17=2 (TWO OR MORE TERMINAL DEVICE TYPES)]**

U_C20. How often do you change between your different types of terminal devices? Would you say…

1 More than 1 time a day

2 Daily

3 2 to 3 times a week

4 Weekly

5 Monthly

6 Rarely

98 DON’T KNOW/NOT SURE [DO NOT READ]

99 REFUSED [DO NOT READ]

**[ASK IF U_C5=1 (YES, CURRENTLY USES PROSTHESIS)]**

U_C21. Is the primary prosthesis and terminal device combination that you use now the same type that you received initially?

**[INTERVIEWER NOTE:** DO NOT READ UNLESS NECESSARY**]**

1 YES

2 NO

98 DON’T KNOW/NOT SURE [DO NOT READ]

99 REFUSED [DO NOT READ]

**[ASK IF U_C5=1 (YES, CURRENTLY USES PROSTHESIS)]**

U_C22. Thinking of the primary prosthesis and terminal device combination you use now, did you receive training to use it?

**[INTERVIEWER NOTE:** DO NOT READ UNLESS NECESSARY**]**

1 YES

2 NO

98 DON’T KNOW/NOT SURE [DO NOT READ]

99 REFUSED [DO NOT READ]

**[ASK IF U_C22=1 (YES, RECEIVED TRAINING)]**

U_C23. How many days or visits of training did you receive?

1 1 to 3

2 4 to 10

3 11 to 20

4 21 to 30

5 More than 30

98 DON’T KNOW/NOT SURE [DO NOT READ]

99 REFUSED [DO NOT READ]

**[ASK IF U_C22=1 (YES, RECEIVED TRAINING)]**

U_C24. Who was the primary person who trained you?

1 A prosthetist

2 A physical or occupational therapist

3 Someone else [Please explain]

98 DON’T KNOW/NOT SURE [DO NOT READ]

99 REFUSED [DO NOT READ]

**[ASK IF U_C24=3 (SOMEONE ELSE)]**

U_C24_ SPECIFY. ___________________________________

**[ASK IF U_C22=1 (YES, RECEIVED TRAINING)]**

U_C25. How would you rate the skills or expertise of the person who trained you to use the prosthesis that you use now? Would you say…

1 Not at all skilled

2 Adequately skilled

3 Highly skilled

98 DON’T KNOW/NOT SURE [DO NOT READ]

99 REFUSED [DO NOT READ]

**[ASK IF U_C5=1 (YES, CURRENTLY USES PROSTHESIS)]**

U_C26. Before you received the prosthesis that you use now, did you discuss or try out different prosthetic options to see which would be best?

**[INTERVIEWER NOTE:** DO NOT READ UNLESS NECESSARY**]**

1 YES

2 NO

98 DON’T KNOW/NOT SURE [DO NOT READ]

99 REFUSED [DO NOT READ]

**Part D: Prosthesis Users: Movement and Sensory Function with Upper Body Tasks**

**[ASK IF U_C5=1 (YES, CURRENTLY USES PROSTHESIS)]**

U_D1. I will now read you a list of statements with which some prosthesis users agree and others disagree. Please rate how you personally feel, as it relates to your **primary** prosthesis and terminal device. This is the combination that you use most often. Please tell me how much you agree with each statement.

**[INTERVIEWER NOTE:** REPEAT RESPONSE OPTIONS AS NECESSARY**]**

|  | Strongly agree | Agree | Disagree | Strongly disagree | DON’T KNOW / NOT APPLICABLE  [DO NOT READ] | REFUSED [DO NOT READ] |
| --- | --- | --- | --- | --- | --- | --- |
| 1. I am satisfied with the way my **primary** prosthesis and terminal device moves. [Would you say Strongly Agree, Agree, Disagree, or Strongly Disagree?] | 1 | 2 | 3 | 4 | 5 | 99 |
| 1. For the next statement, “adequate” means that you do not accidentally crush objects or drop them.   I have an adequate sense of how hard I am gripping with my **primary** prosthesis and terminal device. | 1 | 2 | 3 | 4 | 5 | 99 |
| 1. For the next statement, joints refer to the terminal device position, and positions of any other movable joints of the prosthesis.   I can tell how the joints of my **primary** prosthesis and terminal device are positioned even if I am not looking at them. | 1 | 2 | 3 | 4 | 5 | 99 |
| 1. When I wear my **primary** prosthesis and terminal device, I feel like it is part of my body. | 1 | 2 | 3 | 4 | 5 | 99 |
| 1. My **primary** prosthesis sometimes moves in a way that I do not intend it to. | 1 | 2 | 3 | 4 | 5 | 99 |

**Part E: Prosthesis Users: Satisfaction with Prosthesis**

**[ASK IF U_C5=1 (YES, CURRENTLY USES PROSTHESIS)]**

U_E1. For the next questions, please tell me the extent to which you are satisfied or dissatisfied with each of the different aspects of your primary prosthesis and terminal device.

**[INTERVIEWER NOTE:** REPEAT RESPONSE OPTIONS AS NECESSARY**]**

|  | Very Satisfied | Satisfied | Neither Satisfied nor Dissatisfied | Dissatisfied | Very Dissatisfied | DON’T KNOW/ NOT SURE [DO NOT READ] | REFUSED [DO NOT READ] |
| --- | --- | --- | --- | --- | --- | --- | --- |
| 1. Color [Would you say Very Satisfied, Satisfied, Neither Satisfied nor Dissatisfied, Dissatisfied, or Very Dissatisfied?] | 1 | 2 | 3 | 4 | 5 | 98 | 99 |
| 1. Shape | 1 | 2 | 3 | 4 | 5 | 98 | 99 |
| 1. Noise | 1 | 2 | 3 | 4 | 5 | 98 | 99 |
| 1. Appearance | 1 | 2 | 3 | 4 | 5 | 98 | 99 |
| 1. Weight | 1 | 2 | 3 | 4 | 5 | 98 | 99 |
| 1. Usefulness | 1 | 2 | 3 | 4 | 5 | 98 | 99 |
| 1. Reliability | 1 | 2 | 3 | 4 | 5 | 98 | 99 |
| 1. Fit | 1 | 2 | 3 | 4 | 5 | 98 | 99 |
| 1. Comfort | 1 | 2 | 3 | 4 | 5 | 98 | 99 |
| 1. Overall satisfaction | 1 | 2 | 3 | 4 | 5 | 98 | 99 |

**[ASK IF U_C5=1 (YES, CURRENTLY USES PROSTHESIS)]**

U_E2. For your primary prosthesis and terminal device, how much do you agree or disagree with the following statements?

**[INTERVIEWER NOTE:** REPEAT RESPONSE OPTIONS AS NECESSARY**]**

|  | Strongly agree | Agree | Disagree | Strongly disagree | DON’T KNOW / NOT APPLICABLE  [DO NOT READ] | REFUSED [DO NOT READ] |
| --- | --- | --- | --- | --- | --- | --- |
| 1. I want to change this prosthesis to another type [Would you say Strongly Agree, Agree, Disagree, or Strongly Disagree?] | 1 | 2 | 3 | 4 | 5 | 99 |
| 1. I cannot wear my prosthesis because my **socket** fits poorly | 1 | 2 | 3 | 4 | 5 | 99 |

**[ASK IF U_C5=1 (YES, CURRENTLY USES PROSTHESIS)]**

U_E3. How bothersome is it when you sweat a lot inside your primary prosthesis such as in the sock, liner, or socket on a scale of 0 to 10 with 0 being not at all bothersome and 10 being extremely bothersome.

**[INTERVIEWER NOTE:** DO NOT READ**]**

0 not at all bothersome

1

2

3

4

5

6

7

8

9

10 extremely bothersome

98 DON’T KNOW/NOT SURE [DO NOT READ]

99 REFUSED [DO NOT READ]

**[ASK IF U_C5=1 (YES, CURRENTLY USES PROSTHESIS)]**

U_E4. In the past **12 months**, how many times was your primary prosthesis and terminal device repaired?

1 Never

2 1 time

3 2 to 3 times

4 4 to 5 times

5 6 or more times

98 DON’T KNOW/NOT SURE [DO NOT READ]

99 REFUSED [DO NOT READ]

**[ASK IF U_C5=1 (YES, CURRENTLY USES PROSTHESIS)]**

U_E5. In the past **12 months**, how many times did you visit a prosthetist for adjustments to your socket?

1 Never

2 1 time

3 2 to 3 times

4 4 to 5 times

5 6 or more times

98 DON’T KNOW/NOT SURE [DO NOT READ]

99 REFUSED [DO NOT READ]

**[ASK IF U_C5=1 (YES, CURRENTLY USES PROSTHESIS)]**

U_E6. How many prostheses have you had since your first amputation?

**[INTERVIEWER NOTE:** DO NOT READ UNLESS NECESSARY**]**

1 1

2 2

3 3

4 4

5 5

6 6

7 7

8 8

9 9

10 10 OR MORE

98 DON’T KNOW/NOT SURE [DO NOT READ]

99 REFUSED [DO NOT READ]

**[ASK IF U_C5=1 (YES, CURRENTLY USES PROSTHESIS)]**

U_E7. In the past **12 months**, did your amputation care team or any of your clinical providers ask you what you would like in regards to your prosthetic care?

**[INTERVIEWER NOTE:** DO NOT READ UNLESS NECESSARY**]**

1 YES

2 NO

98 DON’T KNOW/NOT SURE [DO NOT READ]

99 REFUSED [DO NOT READ]

**[ASK IF U_C5=1 (YES, CURRENTLY USES PROSTHESIS)]**

U_E8. For the next questions, please tell me if you strongly agree, agree, disagree, or strongly disagree with each of the following statements about your primary prosthesis and terminal device, and the amputation care you have received in the **past 2 years**.

**[INTERVIEWER NOTE:** For items about the staff, we mean the staff where they receive amputation-related care. If asked about which visit they should be reporting on, it should be in general.**]**

**[INTERVIEWER NOTE:** REPEAT RESPONSE OPTIONS AS NECESSARY**]**

|  | Strongly agree | Agree | Disagree | Strongly disagree | DON’T KNOW / NOT APPLICABLE  [DO NOT READ] | REFUSED [DO NOT READ] |
| --- | --- | --- | --- | --- | --- | --- |
| 1. My prosthesis fits well [Would you say Strongly Agree, Agree, Disagree, or Strongly Disagree?] | 1 | 2 | 3 | 4 | 5 | 99 |
| 1. The weight of my prosthesis is manageable | 1 | 2 | 3 | 4 | 5 | 99 |
| 1. My prosthesis is comfortable throughout the day | 1 | 2 | 3 | 4 | 5 | 99 |
| c.1.My terminal device is appropriately sized for me | 1 | 2 | 3 | 4 | 5 | 99 |
| c.2.Overall, my prosthetic device is appropriately sized for me | 1 | 2 | 3 | 4 | 5 | 99 |
| 1. It is easy to put on my prosthesis | 1 | 2 | 3 | 4 | 5 | 99 |
| 1. My prosthesis looks good | 1 | 2 | 3 | 4 | 5 | 99 |
| 1. I am self-conscious about wearing my prosthesis | 1 | 2 | 3 | 4 | 5 | 99 |
| 1. My prosthesis is durable | 1 | 2 | 3 | 4 | 5 | 99 |
| 1. My clothes are free of wear and tear from my prosthesis | 1 | 2 | 3 | 4 | 5 | 99 |
| 1. My skin is free of abrasions and irritations | 1 | 2 | 3 | 4 | 5 | 99 |
| 1. My prosthesis is pain-free to wear | 1 | 2 | 3 | 4 | 5 | 99 |
| 1. I can afford the out-of-pocket expenses to purchase and maintain my prosthesis | 1 | 2 | 3 | 4 | 5 | 99 |
| 1. I can afford to repair or replace my prosthesis as soon as needed | 1 | 2 | 3 | 4 | 5 | 99 |
| 1. I received an appointment with a prosthetist within a reasonable amount of time **[INTERVIEWER NOTE:** If asked what we mean by “reasonable” - that you thought the wait time to get an appointment was reasonable**]** | 1 | 2 | 3 | 4 | 5 | 99 |
| 1. I was shown the proper level of courtesy and respect by the staff | 1 | 2 | 3 | 4 | 5 | 99 |
| 1. I waited a reasonable amount of time to be seen | 1 | 2 | 3 | 4 | 5 | 99 |
| 1. Clinic staff fully informed me about equipment choices | 1 | 2 | 3 | 4 | 5 | 99 |
| 1. The prosthetist gave me the opportunity to express my concerns regarding my equipment | 1 | 2 | 3 | 4 | 5 | 99 |
| 1. The prosthetist was responsive to my concerns and questions | 1 | 2 | 3 | 4 | 5 | 99 |
| 1. I am satisfied with the training I received in the use and maintenance of my prosthesis | 1 | 2 | 3 | 4 | 5 | 99 |
| 1. The prosthetist discussed problems I might encounter with my equipment | 1 | 2 | 3 | 4 | 5 | 99 |
| 1. The staff coordinated their services with my therapists and doctors | 1 | 2 | 3 | 4 | 5 | 99 |
| 1. I was a partner in decision-making with clinic staff regarding my care and equipment | 1 | 2 | 3 | 4 | 5 | 99 |

**[ASK IF U_C5=1 (YES, CURRENTLY USES PROSTHESIS)]**

U_E8a. How important is it for you to have a prosthesis that does not restrict the type of clothing you wear? Would you say…

1 Very important

2 Somewhat important

3 Not at all important

98 DON’T KNOW/NOT SURE [DO NOT READ]

99 REFUSED [DO NOT READ]

**[ASK IF U_C5=1 (YES, CURRENTLY USES PROSTHESIS)]**

U_E8c. How important is it for you to have a prosthesis that allows you to wear jewelry on your artificial limb, such as a wrist watch, bracelet, or ring? Would you say…

1 Very important

2 Somewhat important

3 Not at all important

98 DON’T KNOW/NOT SURE [DO NOT READ]

99 REFUSED [DO NOT READ]

**Part F: Former Prosthesis**

**[ASK IF U_C1=1 (EVER USED PROSTHESIS)]**

U_F1. Have you ever stopped using a prosthesis?

**[INTERVIEWER NOTE:** DO NOT READ UNLESS NECESSARY**]**

1 YES

2 NO

98 DON’T KNOW/NOT SURE [DO NOT READ]

99 REFUSED [DO NOT READ]

**[ASK IF U_F1=1 (YES, STOPPED USING A PROSTHESIS)]**

U_F2. Were any of the prostheses that you stopped using…

1 Body powered

2 Myoelectric

3 Hybrid

98 DON’T KNOW/NOT SURE [DO NOT READ]

99 REFUSED [DO NOT READ]

**[ASK IF U_F2=1 (BODY POWERED)]**

U_F3. What were the major reasons that you stopped using the body powered prosthesis? Please answer about the last one of this kind that you stopped using.

**[INTERVIEWER NOTE:** REPEAT RESPONSE OPTIONS AS NECESSARY**]**

|  | Yes | No | DON’T KNOW/NOT SURE [DO NOT READ] | REFUSED [DO NOT READ] |
| --- | --- | --- | --- | --- |
| a. It was too heavy or fatiguing to wear [Yes or No?] | 1 | 2 | 98 | 99 |
| b. It didn’t fit or was uncomfortable | 1 | 2 | 98 | 99 |
| c. It was not functional enough | 1 | 2 | 98 | 99 |
| d. It was too much fuss | 1 | 2 | 98 | 99 |
| e. It broke or was unreliable | 1 | 2 | 98 | 99 |
| f. It was not cosmetic | 1 | 2 | 98 | 99 |
| g. It was not intuitive to use | 1 | 2 | 98 | 99 |
| h. Some other reason [IF YES: Please explain] | 1 | 2 | 98 | 99 |

**[ASK IF U_F3h=1 (SOME OTHER REASON)]**

U_F3_SPECIFY. ___________________________________

**[ASK IF U_F2=2 (MYOELECTRIC)]**

U_F4. What were the major reasons that you stopped using the myoelectric prosthesis? Please answer about the last one of this kind that you stopped using.

**[INTERVIEWER NOTE:** REPEAT RESPONSE OPTIONS AS NECESSARY**]**

|  | Yes | No | DON’T KNOW/NOT SURE [DO NOT READ] | REFUSED [DO NOT READ] |
| --- | --- | --- | --- | --- |
| a. It was too heavy or fatiguing to wear [Yes or No?] | 1 | 2 | 98 | 99 |
| b. It didn’t fit or was uncomfortable | 1 | 2 | 98 | 99 |
| c. It was not functional enough | 1 | 2 | 98 | 99 |
| d. It was too much fuss | 1 | 2 | 98 | 99 |
| e. It broke or was unreliable | 1 | 2 | 98 | 99 |
| f. It was not cosmetic | 1 | 2 | 98 | 99 |
| g. It was not intuitive to use | 1 | 2 | 98 | 99 |
| h. Some other reason [IF YES: Please explain] | 1 | 2 | 98 | 99 |

**[ASK IF U_F4h=1 (SOME OTHER REASON)]**

U_F4_SPECIFY. ___________________________________

**[ASK IF U_F2=3 (HYBRID)]**

U_F5. What were the major reasons that you stopped using the hybrid prosthesis? Please answer about the last one of this kind that you stopped using.

**[INTERVIEWER NOTE:** REPEAT RESPONSE OPTIONS AS NECESSARY**]**

|  | Yes | No | DON’T KNOW/NOT SURE [DO NOT READ] | REFUSED [DO NOT READ] |
| --- | --- | --- | --- | --- |
| a. It was too heavy or fatiguing to wear [Yes or No?] | 1 | 2 | 98 | 99 |
| b. It didn’t fit or was uncomfortable | 1 | 2 | 98 | 99 |
| c. It was not functional enough | 1 | 2 | 98 | 99 |
| d. It was too much fuss | 1 | 2 | 98 | 99 |
| e. It broke or was unreliable | 1 | 2 | 98 | 99 |
| f. It was not cosmetic | 1 | 2 | 98 | 99 |
| g. It was not intuitive to use | 1 | 2 | 98 | 99 |
| h. Some other reason [IF YES: Please explain] | 1 | 2 | 98 | 99 |

**[ASK IF U_F5h=1 (SOME OTHER REASON)]**

U_F5_SPECIFY. (Please explain__________________)

**Part G: Pain**

U_G_INTRO The next questions are about pain.

**[ASK ALL]**

U_G1. Over the past 4 weeks, rate how often you experienced back pain. Would you say…

1 Never

2 Only once or twice

3 About once per week

4 2 to 3 times per week

5 4 to 6 times per week

6 Several times every day

7 All the time or almost all the time

98 DON’T KNOW/NOT SURE [DO NOT READ]

99 REFUSED [DO NOT READ]

**[ASK IF U_G1=2-7 (EXPERIENCED ANY PAIN)]**

U_G1a. On average, in the past 4 weeks, how intense was your back pain using a scale of 0 to 10 with 0 being no pain at all and 10 being the worst possible pain?

**[INTERVIEWER NOTE:** DO NOT READ**]**

0 no pain

1

2

3

4

5

6

7

8

9

10 worst possible pain

98 DON’T KNOW/NOT SURE [DO NOT READ]

99 REFUSED [DO NOT READ]

**[ASK ALL]**

U_G2. Over the past 4 weeks, rate how often you had neck pain. Would you say…

1 Never

2 Only once or twice

3 About once per week

4 2 to 3 times per week

5 4 to 6 times per week

6 Several times every day

7 All the time or almost all the time

98 DON’T KNOW/NOT SURE [DO NOT READ]

99 REFUSED [DO NOT READ]

**[ASK IF U_G2=2-7 (EXPERIENCED ANY PAIN)]**

U_G2a. On average, in the past 4 weeks, how intense was your neck pain using a scale of 0 to 10 with 0 being no pain at all and 10 being the worst possible pain?

**[INTERVIEWER NOTE:** DO NOT READ**]**

0 no pain

1

2

3

4

5

6

7

8

9

10 worst possible pain

98 DON’T KNOW/NOT SURE [DO NOT READ]

99 REFUSED [DO NOT READ]

U_G3_INTRO. The next questions are about your phantom limb and residual limb. By phantom limb, we mean feelings of the presence of the limb that you lost. By residual limb, we mean the portion of the limb remaining after the amputation.

**[ASK ALL]**

U_G3. Over the past 4 weeks, rate how often you had pain in your phantom limb or painful phantom limb syndrome. Would you say…

1 Never

2 Only once or twice

3 About once per week

4 2 to 3 times per week

5 4 to 6 times per week

6 Several times every day

7 All the time or almost all the time

98 DON’T KNOW/NOT SURE [DO NOT READ]

99 REFUSED [DO NOT READ]

**[ASK IF U_G3=2-7 (EXPERIENCED ANY PAIN)]**

U_G3a. On average, in the past 4 weeks, how intense was the pain in your phantom limb or painful phantom limb syndrome using a scale of 0 to 10 with 0 being no pain at all and 10 being the worst possible pain?

**[INTERVIEWER NOTE:** DO NOT READ**]**

0 no pain

1

2

3

4

5

6

7

8

9

10 worst possible pain

98 DON’T KNOW/NOT SURE [DO NOT READ]

99 REFUSED [DO NOT READ]

**[ASK IF U_B1=ANY OF 3-99 (NOT AMPUTATED AT SHOULDER OR CHEST LEVEL)]**

U_G4. Over the past 4 weeks, rate how often you had pain in your residual limb. Would you say…

1 Never

2 Only once or twice

3 About once per week

4 2 to 3 times per week

5 4 to 6 times per week

6 Several times every day

7 All the time or almost all the time

98 DON’T KNOW/NOT SURE [DO NOT READ]

99 REFUSED [DO NOT READ]

**[ASK IF U_G4=2-7 (EXPERIENCED ANY PAIN)]**

U_G4a. On average, in the past 4 weeks, how intense was the pain in your residual limb using a scale of 0 to 10 with 0 being no pain at all and 10 being the worst possible pain?

**[INTERVIEWER NOTE:** DO NOT READ**]**

0 no pain

1

2

3

4

5

6

7

8

9

10 worst possible pain

98 DON’T KNOW/NOT SURE [DO NOT READ]

99 REFUSED [DO NOT READ]

**[ASK ALL]**

U_G5. Over the past 4 weeks, rate how often you had pain in your non-amputated limb, including your shoulder, arm or hand. Would you say…

1 Never

2 Only once or twice

3 About once per week

4 2 to 3 times per week

5 4 to 6 times per week

6 Several times every day

7 All the time or almost all the time

98 DON’T KNOW/NOT SURE [DO NOT READ]

99 REFUSED [DO NOT READ]

**[ASK IF U_G5=2-7 (EXPERIENCED ANY PAIN)]**

U_G5a. On average, in the past 4 weeks, how intense was the pain in your non-amputated limb using a scale of 0 to 10 with 0 being no pain at all and 10 being the worst possible pain?

**[INTERVIEWER NOTE:** DO NOT READ**]**

0 no pain

1

2

3

4

5

6

7

8

9

10 worst possible pain

98 DON’T KNOW/NOT SURE [DO NOT READ]

99 REFUSED [DO NOT READ]

**Part H: Difficulty in Activities and Participation**

U_H_INTRO. I will now be asking you some questions about how easy or difficult it is for you to do certain activities. You may answer very easy, easy, difficult, very difficult, or cannot do at all.

**[ASK ALL]**

U_H1. Without a prosthesis, how difficult is it for you to do activities that typically require two hands, such as...

**[INTERVIEWER NOTE:** REPEAT RESPONSE OPTIONS AS NECESSARY**]**

|  | Very easy | Easy | Difficult | Very difficult | Cannot do at all | DON’T KNOW/NOT SURE [DO NOT READ] | REFUSED [DO NOT READ] |
| --- | --- | --- | --- | --- | --- | --- | --- |
| 1. Lift and carry bulky objects like grocery bags or crates that weigh more than 15 pounds? [Would you say Very Easy, Easy, Difficult, Very Difficult, or Cannot Do at All?] | 1 | 2 | 3 | 4 | 5 | 98 | 99 |
| 1. Spread peanut butter on fresh bread? | 1 | 2 | 3 | 4 | 5 | 98 | 99 |
| 1. Do housework such as carrying a laundry basket? | 1 | 2 | 3 | 4 | 5 | 98 | 99 |

**[ASK ALL]**

U_H2. Now please think about how difficult it is for you to do one-handed activities with your residual limb (that is your amputated side) without a prosthesis...

**[INTERVIEWER NOTE:** REPEAT RESPONSE OPTIONS AS NECESSARY**]**

|  | Very easy | Easy | Difficult | Very difficult | Cannot do at all | DON’T KNOW/NOT SURE [DO NOT READ] | REFUSED [DO NOT READ] |
| --- | --- | --- | --- | --- | --- | --- | --- |
| 1. Pick up small objects such as coins and paper clips using your amputated side? [Would you say Very Easy, Easy, Difficult, Very Difficult, or Cannot Do at All?] | 1 | 2 | 3 | 4 | 5 | 98 | 99 |
| 1. Grasp rounded objects, such as cans, using your amputated side? | 1 | 2 | 3 | 4 | 5 | 98 | 99 |

**[ASK IF U_C5=1 (YES, CURRENTLY USES PROSTHESIS)]**

U_H3. Now, I’m going to read you the same set of activities and I’d like you to think about how difficult they are to do using your primary prosthesis and terminal device.

**[INTERVIEWER NOTE:** REPEAT RESPONSE OPTIONS AS NECESSARY**]**

|  | Very easy | Easy | Difficult | Very difficult | Cannot do at all | DON’T KNOW/NOT SURE [DO NOT READ] | REFUSED [DO NOT READ] |
| --- | --- | --- | --- | --- | --- | --- | --- |
| 1. Lift and carry bulky objects like grocery bags or crates that weigh more than 15 pounds? [Would you say Very Easy, Easy, Difficult, Very Difficult, or Cannot Do at All?] | 1 | 2 | 3 | 4 | 5 | 98 | 99 |
| 1. Spread peanut butter on fresh bread? | 1 | 2 | 3 | 4 | 5 | 98 | 99 |
| 1. Do housework such as carrying a laundry basket? | 1 | 2 | 3 | 4 | 5 | 98 | 99 |
| 1. Pick up small objects such as coins and paper clips? | 1 | 2 | 3 | 4 | 5 | 98 | 99 |
| 1. Grasp rounded objects such as cans? | 1 | 2 | 3 | 4 | 5 | 98 | 99 |

**[ASK IF U_C5=1 (YES, CURRENTLY USES PROSTHESIS)]**

U_H4. How comfortable are you in using your prosthesis for physical contact in intimate relationships? Would you say…

1 Very comfortable

2 Comfortable

3 Uncomfortable

4 Very uncomfortable

98 DON’T KNOW/NOT SURE [DO NOT READ]

99 REFUSED [DO NOT READ]

**Part I: Technology Acceptance**

U_I_INTRO. The next questions are about your attitude towards technology.

**[ASK ALL]**

U_I1. How much do you enjoy using high tech gadgets on a scale of 1 to 5, with 1 being Not At All and 5 being Very Much?

**[INTERVIEWER NOTE:** REPEAT SCALE IF NECESSARY**]**

1 NOT AT ALL

2

3

4

5 VERY MUCH

98 DON’T KNOW/NOT SURE [DO NOT READ]

99 REFUSED [DO NOT READ]

**[ASK ALL]**

U_I2. How confident do you feel about your ability to learn to use technology on a scale of 1 to 5 with 1 being Not At All Confident and 5 being Very Confident?

**[INTERVIEWER NOTE:** REPEAT SCALE IF NECESSARY**]**

1 NOT AT ALL CONFIDENT

2

3

4

5 VERY CONFIDENT

98 DON’T KNOW/NOT SURE [DO NOT READ]

99 REFUSED [DO NOT READ]

**Part J: Disability**

U_J_INTRO. The next questions are about your ability to do certain activities.

**[ASK ALL]**

U_J1. Please rate your ability to do the following activities in the **last week**. If you did not have the opportunity to perform an activity in the past week, please make your best estimate of which response would be the most accurate.  It doesn’t matter which hand or arm you use to perform the activity; please answer based on your ability regardless of how you perform the task.

**[INTERVIEWER NOTE:** REPEAT SCALE RESPONSE OPTIONS AS NECESSARY**]**

|  | No  difficulty | Mild difficulty | Moderate difficulty | Severe difficulty | Unable | DON’T KNOW/ NOT SURE [DO NOT READ] | REFUSED [DO NOT READ] |
| --- | --- | --- | --- | --- | --- | --- | --- |
| 1. Open a tight or new jar. [Would you say No Difficulty, Mild Difficulty, Moderate Difficulty, Severe Difficulty, or Unable?] | 1 | 2 | 3 | 4 | 5 | 98 | 99 |
| 1. Do heavy household chores such as wash walls or floors | 1 | 2 | 3 | 4 | 5 | 98 | 99 |
| 1. Carry a shopping bag or briefcase | 1 | 2 | 3 | 4 | 5 | 98 | 99 |
| 1. Wash your back | 1 | 2 | 3 | 4 | 5 | 98 | 99 |
| 1. Use a knife to cut food | 1 | 2 | 3 | 4 | 5 | 98 | 99 |
| 1. Recreational activities in which you take some force or impact through your arm, shoulder or hand such as golf, hammering, or tennis | 1 | 2 | 3 | 4 | 5 | 98 | 99 |

**[ASK ALL]**

U_J2. During the past week, to what extent has your arm, shoulder or hand problem interfered with your normal social activities with family, friends, neighbors or groups? Would you say…

1 Not at all

2 Slightly

3 Moderately

4 Quite a bit

5 Extremely

98 DON’T KNOW/NOT SURE [DO NOT READ]

99 REFUSED [DO NOT READ]

**[ASK ALL]**

U_J3. During the past week, were you limited in your work or other regular daily activities as a result of your arm, shoulder or hand problem? Would you say…

1 Not limited at all

2 Slightly limited

3 Moderately limited

4 Very limited

5 Unable

98 DON’T KNOW/NOT SURE [DO NOT READ]

99 REFUSED [DO NOT READ]

**[ASK ALL]**

U_J4. During the past week, how much difficulty have you had sleeping because of the pain in your arm, shoulder or hand? Would you say…

1 No difficulty

2 Mild difficulty

3 Moderate difficulty

4 Severe difficulty

5 So much difficulty that I can’t sleep

98 DON’T KNOW/NOT SURE [DO NOT READ]

99 REFUSED [DO NOT READ]

**[ASK ALL]**

U_J5. Please rate the severity of the following symptoms in the **last week**.

|  | None | Mild | Moderate | Severe | Extreme | DON’T KNOW/ NOT SURE [DO NOT READ] | REFUSED [DO NOT READ] |
| --- | --- | --- | --- | --- | --- | --- | --- |
| 1. Arm, shoulder or hand pain. [Would you say None, Mild, Moderate, Severe, or Extreme?] | 1 | 2 | 3 | 4 | 5 | 98 | 99 |
| 1. Tingling (pins and needles) in your arm, shoulder or hand. | 1 | 2 | 3 | 4 | 5 | 98 | 99 |

**Part K: Health and Quality of Life**

U_K_INTRO. The next questions are about your overall health and quality of life.

**[ASK ALL]**

U_K1. The first question is about your health. In general, would you say your health is…

1 Excellent

2 Very good

3 Good

4 Fair

5 Poor

98 DON’T KNOW/NOT SURE [DO NOT READ]

99 REFUSED [DO NOT READ]

U_K2_INTRO. The next few questions are about activities you might do during a typical day. For each of the following types of activities, please tell me if your health now limits you a lot, limits you a little, or does not limit you at all.

**[ASK ALL]**

U_K2. **Moderate activities** such as moving a table, pushing a vacuum cleaner, bowling, or playing golf? Because of your health, are you…

1 Limited a lot

2 Limited a little

3 Not limited at all

4 DO NOT DO ACTIVITY [DO NOT READ]

97 DO NOT HAVE HEALTH PROBLEMS/HEALTH IS FINE [DO NOT READ]

98 DON’T KNOW/NOT SURE [DO NOT READ]

99 REFUSED [DO NOT READ]

**[ASK IF U_K2=4 (DO NOT DO ACTIVITY)]**

U_K3. Is that because of your health?

**[INTERVIEWER NOTE:** DO NOT READ UNLESS NECESSARY**]**

1 YES

2 NO

98 DON’T KNOW/NOT SURE [DO NOT READ]

99 REFUSED [DO NOT READ]

**[IF U_K3=1, CODE U_K2=1; IF U_K3=2, CODE U_K2=3; IF U_K3=98 OR 99, DO NOT RECODE U_K2]**

**[ASK ALL]**

U_K4. **Climbing several flights of stairs?** Because of your health, are you…

1 Limited a lot

2 Limited a little

3 Not limited at all

4 DO NOT DO ACTIVITY [DO NOT READ]

97 DO NOT HAVE HEALTH PROBLEMS/HEALTH IS FINE [DO NOT READ]

98 DON’T KNOW/NOT SURE [DO NOT READ]

99 REFUSED [DO NOT READ]

**[ASK IF U_K4=4 (DO NOT DO ACTIVITY)]**

U_K5. Is that because of your health?

**[INTERVIEWER NOTE:** DO NOT READ UNLESS NECESSARY**]**

1 YES

2 NO

98 DON’T KNOW/NOT SURE [DO NOT READ]

99 REFUSED [DO NOT READ]

**[IF U_K5=1, CODE U_K4=1; IF U_K5=2, CODE U_K4=3; IF U_K5=98 OR 99, DO NOT RECODE U_K4]**

**[ASK ALL]**

U_K6. During the past 4 weeks, how much of the time have you **accomplished less than you would like** **with your work or other regular activities** as a result of your physical health? Would you say…

1 None of the time

2 A little of the time

3 Some of the time

4 Most of the time

5 All of the time

97 DO NOT HAVE HEALTH PROBLEMS/HEALTH IS FINE [DO NOT READ]

98 DON’T KNOW/NOT SURE [DO NOT READ]

99 REFUSED [DO NOT READ]

**[ASK ALL]**

U_K7. During the past 4 weeks, how much were you **limited in the kind of work or other activities** as a result of your physical health? Would you say…

1 None of the time

2 A little of the time

3 Some of the time

4 Most of the time

5 All of the time

97 DO NOT HAVE HEALTH PROBLEMS/HEALTH IS FINE [DO NOT READ]

98 DON’T KNOW/NOT SURE [DO NOT READ]

99 REFUSED [DO NOT READ]

U_K8_INTRO. The next few questions are about your emotions and daily activities.

**[ASK ALL]**

U_K8. During the past 4 weeks, how much of the time have you **accomplished less than you would like** **with your work or other regular activities** as a result of any emotional problems, such as feeling depressed or anxious? **[INTERVIEWER NOTE: REPEAT SCALE AS NECESSARY]**

1 None of the time

2 A little of the time

3 Some of the time

4 Most of the time

5 All of the time

97 DO NOT HAVE EMOTIONAL PROBLEMS [DO NOT READ]

98 DON’T KNOW/NOT SURE [DO NOT READ]

99 REFUSED [DO NOT READ]

**[ASK ALL]**

U_K9. During the past 4 weeks, how much of the time did you not do work or other activities as **carefully** **as usual** as a result of any emotional problems, such as feeling depressed or anxious? **[INTERVIEWER NOTE: REPEAT SCALE AS NECESSARY]**

1 None of the time

2 A little of the time

3 Some of the time

4 Most of the time

5 All of the time

97 DO NOT HAVE EMOTIONAL PROBLEMS [DO NOT READ]

98 DON’T KNOW/NOT SURE [DO NOT READ]

99 REFUSED [DO NOT READ]

**[ASK ALL]**

U_K10. During the past 4 weeks, how much did **pain** interfere with your normal work, including both work outside the home and house work? Would you say…

1 Not at all

2 A little bit

3 Moderately

4 Quite a bit

5 Extremely

97 DO NOT HAVE PAIN [DO NOT READ]

98 DON’T KNOW/NOT SURE [DO NOT READ]

99 REFUSED [DO NOT READ]

U_K11_INTRO. The next questions are about how you feel and how things have been with you during the past 4 weeks. For each question, please give the one answer that comes closest to the way you have been feeling.

**[ASK ALL]**

U_K11. How much of the time during the past 4 weeks have you felt **calm and peaceful**? Would you say…

1 All of the time

2 Most of the time

3 A good bit of the time

4 Some of the time

5 A little of the time

6 None of the time

98 DON’T KNOW/NOT SURE [DO NOT READ]

99 REFUSED [DO NOT READ]

**[ASK ALL]**

U_K12. How much of the time during the past 4 weeks did you have **a lot of energy**?

**[INTERVIEWER NOTE: REPEAT SCALE AS NECESSARY]**

1 All of the time

2 Most of the time

3 A good bit of the time

4 Some of the time

5 A little of the time

6 None of the time

98 DON’T KNOW/NOT SURE [DO NOT READ]

99 REFUSED [DO NOT READ]

**[ASK ALL]**

U_K13. How much of the time during the past 4 weeks have you felt **downhearted and blue**? **[INTERVIEWER NOTE: REPEAT SCALE AS NECESSARY]**

1 All of the time

2 Most of the time

3 A good bit of the time

4 Some of the time

5 A little of the time

6 None of the time

98 DON’T KNOW/NOT SURE [DO NOT READ]

99 REFUSED [DO NOT READ]

**[ASK ALL]**

U_K14. During the past 4 weeks, how much of the time have your **physical health or emotional problems** interfered with your social activities like visiting friends, relatives, etc.? Would you say…

1 All of the time

2 Most of the time

3 Some of the time

4 A little of the time

5 None of the time

97 DO NOT HAVE PHYSICAL OR EMOTIONAL PROBLEMS [DO NOT READ]

98 DON’T KNOW/NOT SURE [DO NOT READ]

99 REFUSED [DO NOT READ]

**[ASK ALL]**

U_K15. **Compared to one year ago,** how would you rate your physical health in general now? Would you say…

1 Much better

2 Slightly better

3 About the same

4 Slightly worse

5 Much worse

98 DON’T KNOW/NOT SURE [DO NOT READ]

99 REFUSED [DO NOT READ]

**[ASK ALL]**

U_K16. **Compared to one year ago,** how would you rate your emotional problems such as feeling anxious, depressed, or irritable now? Would you say…

1 Much better

2 Slightly better

3 About the same

4 Slightly worse

5 Much worse

97 DO NOT HAVE EMOTIONAL PROBLEMS [DO NOT READ]

98 DON’T KNOW/NOT SURE [DO NOT READ]

99 REFUSED [DO NOT READ]

**[ASK ALL]**

U_K17. Please answer how much you agree or disagree with the statement: I am self-conscious about my amputation. Would you say…

1 Strongly agree

2 Agree

3 Disagree

4 Strongly disagree

98 DON’T KNOW/NOT SURE [DO NOT READ]

99 REFUSED [DO NOT READ]

**[ASK ALL]**

U_K18. Do you currently experience flashbacks or nightmares about your amputation?

**[INTERVIEWER NOTE:** DO NOT READ UNLESS NECESSARY**]**

1 YES

2 NO

98 DON’T KNOW/NOT SURE [DO NOT READ]

99 REFUSED [DO NOT READ]

**Part L: Quality of Care and Clinical Practice Guidelines**

U_L_INTRO. The next questions are about amputation care.

**[ASK ALL]**

U_L1. Which of the following places have you **ever** gone to for amputation-related care?

**[INTERVIEWER NOTE:** REPEAT RESPONSE OPTIONS AS NECESSARY**]**

|  | Yes | No | DON’T KNOW/NOT SURE [DO NOT READ] | REFUSED [DO NOT READ] |
| --- | --- | --- | --- | --- |
| a. VA medical center [Yes or No?] | 1 | 2 | 98 | 99 |
| b. Local prosthetist office | 1 | 2 | 98 | 99 |
| c. Non-VA health care center or hospital | 1 | 2 | 98 | 99 |
| d. Department of Defense medical center | 1 | 2 | 98 | 99 |
| e. Someplace else [IF YES: Please explain] | 1 | 2 | 98 | 99 |

**[ASK IF U_L1e=1 (SOMEPLACE ELSE)]**

U_L1_SPECIFY. __________________________________

**[ASK ALL]**

U_L2. Have you **ever** been to a VA Amputation Clinic?

**[INTERVIEWER NOTE:** DO NOT READ UNLESS NECESSARY**]**

1 YES

2 NO

3 NOT SURE

99 REFUSED [DO NOT READ]

**[ASK IF U_L2=1 (YES)]**

U_L3. What was the year of your last visit to a VA Amputation Clinic?

[**INTERVIEWER NOTE:** ENTER 98 FOR DON’T KNOW AND 99 FOR REFUSED]

YEAR: ________

**[ASK ALL]**

U_L4. Have you **ever** been to a Department of Defense Amputation Clinic?

**[INTERVIEWER NOTE:** DO NOT READ UNLESS NECESSARY**]**

1 YES

2 NO

3 NOT SURE

99 REFUSED [DO NOT READ]

**[ASK IF U_L4=1 (YES)]**

U_L5. What was the year of your last visit to a Department of Defense Amputation Clinic?

**[INTERVIEWER NOTE:** ENTER 98 FOR DON’T KNOW AND 99 FOR REFUSED]

YEAR: ________

**[ASK ALL]**

U_L6. In the past **12 months**, did any clinician test your strength, flexibility or ability to do daily activities? Please do not include a clinician you may have seen during a research study.

**[INTERVIEWER NOTE:** DO NOT READ UNLESS NECESSARY**]**

1 YES

2 NO

98 DON’T KNOW/NOT SURE [DO NOT READ]

99 REFUSED [DO NOT READ]

**[ASK ALL]**

U_L7. In the past **12 months**, did you receive any amputation-related care?

**[INTERVIEWER NOTE:** DO NOT READ UNLESS NECESSARY**]**

1 YES

2 NO

98 DON’T KNOW/NOT SURE [DO NOT READ]

99 REFUSED [DO NOT READ]

**[ASK IF U_L7=1 (YES)]**

U_L8. Which type of providers have been involved in your amputation care in the past **12 months**?

**[INTERVIEWER NOTE:** REPEAT RESPONSE OPTIONS AS NECESSARY**]**

|  | Yes | No | DON’T KNOW/NOT SURE [DO NOT READ] | REFUSED [DO NOT READ] |
| --- | --- | --- | --- | --- |
| a. A prosthetist [Yes or No?] | 1 | 2 | 98 | 99 |
| b. A primary care doctor | 1 | 2 | 98 | 99 |
| c. A specialist physician, such as a neurologist, physiatrist, or orthopedic surgeon | 1 | 2 | 98 | 99 |
| d. A mental and behavioral health specialist such as a psychologist, psychiatrist, psychotherapist, or mental health counselor | 1 | 2 | 98 | 99 |
| e. A case manager or social worker | 1 | 2 | 98 | 99 |
| f. A nurse who is not a case manager | 1 | 2 | 98 | 99 |
| g. An occupational or physical therapist | 1 | 2 | 98 | 99 |
| h. A driver rehabilitation therapist **[INTERVIEWER NOTE:** A Driver Rehabilitation Therapist is someone who works with the patient to help them return to driving an automobile.**]** | 1 | 2 | 98 | 99 |
| i. A recreation therapist | 1 | 2 | 98 | 99 |
| j. Someone else [IF YES: Please explain] | 1 | 2 | 98 | 99 |

**[ASK IF U_L8j=1 (SOMEONE ELSE)]**

U_L8_SPECIFY. __________________________________

**[ASK IF U_L7=1 (YES)]**

U_L9. In the past **12 months**, have you discussed goals related to your amputation care with any of your clinical providers?

**[INTERVIEWER NOTE:** DO NOT READ UNLESS NECESSARY**]**

1 YES

2 NO

98 DON’T KNOW/NOT SURE [DO NOT READ]

99 REFUSED [DO NOT READ]

**[ASK IF U_L9=1**

U_L10. In the past **12 months**, have you worked with clinical providers to develop a plan to accomplish your goals?

**[INTERVIEWER NOTE:** DO NOT READ UNLESS NECESSARY**]**

1 YES

2 NO

98 DON’T KNOW/NOT SURE [DO NOT READ]

99 REFUSED [DO NOT READ]

**[ASK ALL]**

U_L11. In the past **12 months**, did you have a prosthetic or amputation check-up, either by phone or in-person at the VA?

[INTERVIEWER NOTE: RESPONDENT CANNOT SELECT BOTH YES AND NO]

1 Yes, in person

2 Yes, by telephone

3 No

98 DON’T KNOW/NOT SURE [DO NOT READ]

99 REFUSED [DO NOT READ]

**[ASK IF U_L11=1 (YES, IN PERSON)]**

U_L12. Who was involved with this in-person appointment at the VA?

**[INTERVIEWER NOTE:** REPEAT RESPONSE OPTIONS AS NECESSARY**]**

|  | Yes | No | DON’T KNOW/NOT SURE [DO NOT READ] | REFUSED [DO NOT READ] |
| --- | --- | --- | --- | --- |
| a. A prosthetist [Yes or No?] | 1 | 2 | 98 | 99 |
| b. A physical therapist or occupational therapist | 1 | 2 | 98 | 99 |
| c. A physician | 1 | 2 | 98 | 99 |
| d. A nurse | 1 | 2 | 98 | 99 |
| e. Another clinician [IF YES: Please explain] | 1 | 2 | 98 | 99 |

**[ASK IF U_L12e=1 (ANOTHER CLINICIAN)]**

U_L12_SPECIFY. _________________________________

**[ASK ALL]**

U_L13. Do any of your providers contact you to check in to see how you are doing outside of an appointment?

**[INTERVIEWER NOTE:** DO NOT READ UNLESS NECESSARY**]**

1 YES

2 NO

98 DON’T KNOW/NOT SURE [DO NOT READ]

99 REFUSED [DO NOT READ]

U_L14_INTRO. The next questions are about your family or other caregivers who were involved in the management of your amputation, such as attending visits with the prosthetist, discussing technology with you, or assisting in obtaining insurance reimbursement.

**[ASK ALL]**

U_L14. Were your family or other caregivers involved in the management of your amputation immediately after your amputation?

**[INTERVIEWER NOTE:** DO NOT READ UNLESS NECESSARY**]**

1 YES

2 NO

98 DON’T KNOW/NOT SURE [DO NOT READ]

99 REFUSED [DO NOT READ]

**[ASK ALL]**

U_L15. Were your family or other caregivers involved in the management of your amputation in the past **12** **months**?

**[INTERVIEWER NOTE:** DO NOT READ UNLESS NECESSARY**]**

1 YES

2 NO

98 DON’T KNOW/NOT SURE [DO NOT READ]

99 REFUSED [DO NOT READ]

**[ASK ALL]**

U_L16. Did you move in the past **12 months**?

**[INTERVIEWER NOTE:** DO NOT READ UNLESS NECESSARY**]**

1 YES

2 NO

98 DON’T KNOW/NOT SURE [DO NOT READ]

99 REFUSED [DO NOT READ]

**[ASK IF U_L16=1 (YES)]**

U_L17. Did any of your providers help you to coordinate with new providers when you moved?

**[INTERVIEWER NOTE:** DO NOT READ UNLESS NECESSARY**]**

1 YES

2 NO

3 Not applicable; did not need to change providers

98 DON’T KNOW/NOT SURE [DO NOT READ]

99 REFUSED [DO NOT READ]

**[ASK ALL]**

U_L18. Do you typically experience amputation-related pain in your stump, phantom limb, or sound arm?

**[INTERVIEWER NOTE:** DO NOT READ UNLESS NECESSARY**]**

1 YES

2 NO

98 DON’T KNOW/NOT SURE [DO NOT READ]

99 REFUSED [DO NOT READ]

**[ASK IF U_L18=1 (YES, EXPERIENCE PAIN)]**

U_L19. Do you feel that your pain is well managed?

**[INTERVIEWER NOTE:** DO NOT READ UNLESS NECESSARY**]**

1 YES

2 NO

98 DON’T KNOW/NOT SURE [DO NOT READ]

99 REFUSED [DO NOT READ]

**[ASK IF U_L18=1 (YES, EXPERIENCE PAIN)]**

U_L20. Do you manage your amputation related pain using any type of medication?

**[INTERVIEWER NOTE:** DO NOT READ UNLESS NECESSARY**]**

1 YES

2 NO

98 DON’T KNOW/NOT SURE [DO NOT READ]

99 REFUSED [DO NOT READ]

**[ASK IF U_L18=1 (YES, EXPERIENCE PAIN)]**

U_L21. Do you manage your amputation related pain using any other strategy such as exercise, acupuncture, or hobbies?

**[INTERVIEWER NOTE:** DO NOT READ UNLESS NECESSARY**]**

1 YES

2 NO

98 DON’T KNOW/NOT SURE [DO NOT READ]

99 REFUSED [DO NOT READ]

**[ASK ALL]**

U_L22. Following your amputation did you have a visit from a peer visitor who is also an amputee?

**[INTERVIEWER NOTE:** DO NOT READ UNLESS NECESSARY**]**

1 YES

2 NO

3 Don’t remember

99 REFUSED [DO NOT READ]

**[ASK ALL]**

U_L23. In the past **12 months**, have you received any information about the management of your amputation such as information about new technologies, surgical techniques, and rehabilitation procedures?

**[INTERVIEWER NOTE:** DO NOT READ UNLESS NECESSARY**]**

1 YES

2 NO

98 DON’T KNOW/NOT SURE [DO NOT READ]

99 REFUSED [DO NOT READ]

**Part M: Risk Benefit Questions**

U_M_INTRO. I am now going to briefly describe three new upper limb prostheses and technologies that are in development. This is for research purposes only - we are not trying to market or sell anything. For each new technology, I will ask if you would be willing to undergo surgery for it, and if so, I will ask you some questions about the risks and benefits associated with that technology.

**[ASK ALL]**

U_M1. Would you consider undergoing surgery to have a prosthesis that would **restore a sense of touch**?

**[INTERVIEWER NOTE:** DO NOT READ UNLESS NECESSARY**]**

1 YES

2 NO

3 NOT SURE

99 REFUSED [DO NOT READ]

**[ASK IF U_M1=1 (YES) OR 3 (NOT SURE)]**

U_M2. How important are the following benefits in your decision to consider undergoing surgery for a device that **restores a sense of touch?**

**[INTERVIEWER NOTE:** REPEAT RESPONSE OPTIONS AS NECESSARY**]**

|  | **Very important** | **Somewhat important** | **Not at all important** | DON’T KNOW/NOT SURE [DO NOT READ] | REFUSED [DO NOT READ] |
| --- | --- | --- | --- | --- | --- |
| 1. A sense of touch that feels natural [Would you say Very Important, Somewhat Important, or Not at All Important?] | 1 | 2 | 3 | 98 | 99 |
| 1. The ability to do more activities with the prosthesis | 1 | 2 | 3 | 98 | 99 |
| 1. Getting a durable and reliable prosthesis that seldom needs repair | 1 | 2 | 3 | 98 | 99 |
| 1. A prosthesis that can get wet or dirty | 1 | 2 | 3 | 98 | 99 |
| 1. A comfortable prosthesis | 1 | 2 | 3 | 98 | 99 |
| 1. A lightweight prosthesis | 1 | 2 | 3 | 98 | 99 |
| 1. A prosthesis that can be used to lift more than 20 pounds | 1 | 2 | 3 | 98 | 99 |

**[ASK IF U_M1=1 (YES) OR 3 (NOT SURE)]**

U_M3. Would you be willing to accept the following risks and inconveniences to undergo a surgery that would let you use a prosthesis that **restores a sense of touch?**

**[INTERVIEWER NOTE:** REPEAT RESPONSE OPTIONS AS NECESSARY**]**

|  | **Yes** | **No** | **Maybe** | DON’T KNOW/NOT SURE [DO NOT READ] | REFUSED [DO NOT READ] |
| --- | --- | --- | --- | --- | --- |
| 1. Surgery would require an overnight stay [Would you say Yes, No, or Maybe?] | 1 | 2 | 3 | 98 | 99 |
| 1. Risk of infection that would require antibiotics | 1 | 2 | 3 | 98 | 99 |
| 1. Risk of serious infection that would require removing the device | 1 | 2 | 3 | 98 | 99 |
| 1. Long-term risks, such as chronic pain, loss of some nerve function, or device failure requiring it to be removed | 1 | 2 | 3 | 98 | 99 |
| 1. Pain or weakness during the recovery from surgery of about 1 month | 1 | 2 | 3 | 98 | 99 |
| 1. Short-term restrictions on movement and exercise for up to 1 month | 1 | 2 | 3 | 98 | 99 |

**[ASK ALL]**

U_M4. Would you consider undergoing surgery to have a prosthesis that would give you **more control over finger movements, grasps, or wrist motions**?

**[INTERVIEWER NOTE:** DO NOT READ UNLESS NECESSARY**]**

1 YES

2 NO

3 NOT SURE

99 REFUSED [DO NOT READ]

**[ASK IF U_M4=1 (YES) OR 3 (NOT SURE)]**

U_M5. How important are the following benefits in your decision to consider undergoing surgery to have a device that gives you **more control over finger movements, grasps, or wrist motions**?

**[INTERVIEWER NOTE:** REPEAT RESPONSE OPTIONS AS NECESSARY**]**

|  | **Very important** | **Somewhat important** | **Not at all important** | DON’T KNOW/NOT SURE [DO NOT READ] | REFUSED [DO NOT READ] |
| --- | --- | --- | --- | --- | --- |
| 1. The ability to directly control the prosthesis finger movements, grasps, or wrist motions [Would you say Very Important, Somewhat Important, or Not at All Important?] | 1 | 2 | 3 | 98 | 99 |
| 1. A device that allows for better control over the speed of the prosthesis movement | 1 | 2 | 3 | 98 | 99 |
| 1. The ability to do more activities with the prosthesis | 1 | 2 | 3 | 98 | 99 |
| 1. Getting a durable and reliable prosthesis that seldom needs repair | 1 | 2 | 3 | 98 | 99 |
| 1. A prosthesis that can get wet or dirty | 1 | 2 | 3 | 98 | 99 |
| 1. A comfortable prosthesis | 1 | 2 | 3 | 98 | 99 |
| 1. A lightweight prosthesis | 1 | 2 | 3 | 98 | 99 |
| 1. A prosthesis that can be used to lift more than 20 pounds | 1 | 2 | 3 | 98 | 99 |

**[ASK IF U_M4=1 (YES) OR 3 (NOT SURE)]**

U_M6. Would you be willing to accept the following risks and inconveniences to undergo a surgery that would let you use a prosthesis to gain **more control over finger movements, grasps, or wrist motions**?

**[INTERVIEWER NOTE:** REPEAT RESPONSE OPTIONS AS NECESSARY**]**

|  | **Yes** | **No** | **Maybe** | DON’T KNOW/NOT SURE [DO NOT READ] | REFUSED [DO NOT READ] |
| --- | --- | --- | --- | --- | --- |
| 1. Surgery would require an overnight stay [Would you say Yes, No, or Maybe?] | 1 | 2 | 3 | 98 | 99 |
| 1. Risk of infection that would require antibiotics | 1 | 2 | 3 | 98 | 99 |
| 1. Risk of serious infection that would require removing the device | 1 | 2 | 3 | 98 | 99 |
| 1. Long-term risks, such as chronic pain, loss of some nerve function, or device failure requiring it to be removed | 1 | 2 | 3 | 98 | 99 |
| 1. Pain or weakness during the recovery from surgery of about 1 month | 1 | 2 | 3 | 98 | 99 |
| 1. Short-term restrictions on movement and exercise for up to 1 month | 1 | 2 | 3 | 98 | 99 |
| 1. Skin irritation or breakdown from the socket or components socket | 1 | 2 | 3 | 98 | 99 |

**[ASK ALL]**

U_M7. Osseointegration is a surgery that would implant a metal rod directly into the bone and allow a prosthesis to be attached to it. A socket and harness would no longer be needed. The metal rod would be visible when the prosthesis is not in use and the skin around the rod will need to be cleaned every day to avoid infections. Would you consider having osseointegration surgery?

**[INTERVIEWER NOTE:** DO NOT READ UNLESS NECESSARY**]**

1 YES

2 NO

3 NOT SURE

99 REFUSED [DO NOT READ]

**[ASK IF U_M7=1 (YES) OR 3 (NOT SURE)]**

U_M8. How important are the following benefits in your decision to consider undergoing **osseointegration** surgery?

**[INTERVIEWER NOTE:** REPEAT RESPONSE OPTIONS AS NECESSARY**]**

|  | **Very important** | **Somewhat important** | **Not at all important** | DON’T KNOW/NOT SURE [DO NOT READ] | REFUSED [DO NOT READ] |
| --- | --- | --- | --- | --- | --- |
| 1. A sense of touch that feels natural [Would you say Very Important, Somewhat Important, or Not at All Important?] | 1 | 2 | 3 | 98 | 99 |
| 1. The ability to do more activities with the prosthesis | 1 | 2 | 3 | 98 | 99 |
| 1. Getting a durable and reliable prosthesis that seldom needs repair | 1 | 2 | 3 | 98 | 99 |
| 1. A prosthesis that can get wet or dirty | 1 | 2 | 3 | 98 | 99 |
| 1. A comfortable prosthesis | 1 | 2 | 3 | 98 | 99 |
| 1. A lightweight prosthesis | 1 | 2 | 3 | 98 | 99 |
| 1. A prosthesis that can be used to lift more than 20 pounds | 1 | 2 | 3 | 98 | 99 |

**[ASK IF U_M7=1 (YES) OR 3 (NOT SURE)]**

U_M9. Would you be willing to accept the following risks and inconveniences to undergo surgery to have an **osseointegrated prosthesis**?

**[INTERVIEWER NOTE:** REPEAT RESPONSE OPTIONS AS NECESSARY**]**

|  | **Yes** | **No** | **Maybe** | DON’T KNOW/NOT SURE [DO NOT READ] | REFUSED [DO NOT READ] |
| --- | --- | --- | --- | --- | --- |
| 1. Risk of infection that would require hospitalization and IV antibiotics for 6 weeks [Would you say Yes, No, or Maybe?] | 1 | 2 | 3 | 98 | 99 |
| 1. Risk of serious infection that would require removing the device | 1 | 2 | 3 | 98 | 99 |
| 1. Risk of bone breaking that would require surgery to remove the device | 1 | 2 | 3 | 98 | 99 |
| 1. Long-term risks, such as chronic pain, loss of some nerve function, or device failure requiring it to be removed | 1 | 2 | 3 | 98 | 99 |
| 1. Pain or weakness during the recovery from surgery of about 1 month | 1 | 2 | 3 | 98 | 99 |
| 1. Short-term restrictions on movement and exercise for up to 1 month | 1 | 2 | 3 | 98 | 99 |
| 1. Up to 6 months of physical therapy to train you to use the prosthesis and use it for lifting and carrying | 1 | 2 | 3 | 98 | 99 |

**[ASK IF SAMPLE VARIABLE SEX = FEMALE]**

U_M10. There has been very little research about women with an upper limb amputation. Is there anything you would like to tell us about your experience that that we haven’t asked in this survey?

**[INTERVIEWER NOTE:** IF RESPONDENT HAS NO COMMENTS, **PROMPT**: Any additional detail you can provide about your experience as a woman with an upper limb amputation will be helpful for the researchers.**]**

**­­____________________________________________________________________**

**Part N: Questions about You**

U_N_INTRO. The final questions are about you. These questions are for analysis purposes only.

**[ASK ALL]**

U_N1. What is your age?

[INTERVIEWER NOTE: ENTER 998 FOR DON’T KNOW AND 999 FOR REFUSED]

**[ASK ALL]**

U_N2. What is your sex?

**[INTERVIEWER NOTE:** DO NOT READ UNLESS NECESSARY**]**

1 MALE

2 FEMALE

98 DON’T KNOW/NOT SURE [DO NOT READ]

99 REFUSED [DO NOT READ]

**[ASK ALL]**

U_N3. What is your current military status?

1 Veteran

2 Civilian

3 Active Duty

98 DON’T KNOW/NOT SURE [DO NOT READ]

99 REFUSED [DO NOT READ]

**[ASK ALL]**

U_N4. What is your current employment status?

1 Employed full-time

2 Employed part-time

3 Student

4 Retired, but was employed after my amputation

5 Retired, but was NOT employed after my amputation

6 On medical leave

7 Other [Please explain]

98 DON’T KNOW/NOT SURE [DO NOT READ]

99 REFUSED [DO NOT READ]

**[ASK IF U_N4=7 (OTHER)]**

U_N4_SPECIFY ________________________________

**[ASK ALL]**

U_N5. What is your marital status? Are you…

1 Married or living together

2 Divorced or separated

3 Widowed

4 Never married

98 DON’T KNOW/NOT SURE [DO NOT READ]

99 REFUSED [DO NOT READ]

**[ASK ALL]**

U_N6. Do you have children?

**[INTERVIEWER NOTE:** DO NOT READ UNLESS NECESSARY**]**

1 YES

2 NO

98 DON’T KNOW/NOT SURE [DO NOT READ]

99 REFUSED [DO NOT READ]

**[ASK ALL]**

U_N7a. What is your race? I will read you a list of choices. You may choose one or more.

**[**CHECK ALL THAT APPLY**]**

1 White or Caucasian

2 Black or African-American

3 Asian

4 American Indian or Alaska Native

5 Native Hawaiian or Pacific Islander

6 Other [Please explain]

98 DON’T KNOW/NOT SURE [DO NOT READ]

99 REFUSED [DO NOT READ]

**[ASK IF U_N7A=6 (OTHER)]**

U_N7a_SPECIFY. __________________________________

**[ASK ALL]**

U_N7b. Are you of Hispanic or Latino origin or descent?

**[INTERVIEWER NOTE:** DO NOT READ UNLESS NECESSARY**]**

1 Yes, Hispanic or Latino

2 No, not Hispanic or Latino

98 DON’T KNOW/NOT SURE [DO NOT READ]

99 REFUSED [DO NOT READ]

**Part O: Follow-Up Permission**

**[ASK ALL]**

U_O1. Thank you for taking the time to complete this survey. May we contact you in the future to see if you are interested in participating in other study activities?

**[INTERVIEWER NOTE:** DO NOT READ UNLESS NECESSARY**]**

1 Yes

2 No

98 DON’T KNOW/NOT SURE [DO NOT READ]

99 REFUSED [DO NOT READ]
